# Single-Atom Cu Anchored on Carbon Nitride as a Bifunctional Glucose Oxidase and Peroxidase Nanozyme for Antibacterial Therapy

**DOI:** 10.1021/acsnano.4c12348

**Published:** 2025-03-14

**Authors:** Fan Wu, Yaran Wang, Yuanfeng Li, Linqi Shi, Lu Yuan, Yijin Ren, Henny C. van der Mei, Yong Liu

**Affiliations:** 1 Translational Medicine Laboratory, the First Affiliated Hospital of Wenzhou Medical University, Wenzhou, Zhejiang 325035, China; 2 Department of Biomaterials & Biomedical Technology, University of Groningen and University Medical Center Groningen, Antonius Deusinglaan 1, Groningen 9713 AV, The Netherlands; 3 State Key Laboratory of Medicinal Chemical Biology, Key Laboratory of Functional Polymer Materials, Ministry of Education, Institute of Polymer Chemistry, College of Chemistry, Nankai University, Tianjin 300071, China; 4 Department of Orthodontics, University of Groningen and University Medical Center Groningen, Hanzeplein 1, Groningen 9700 RB, The Netherlands

**Keywords:** single-atom nanozyme, cascade reaction, bacterial infection, biofilm, wound dressing

## Abstract

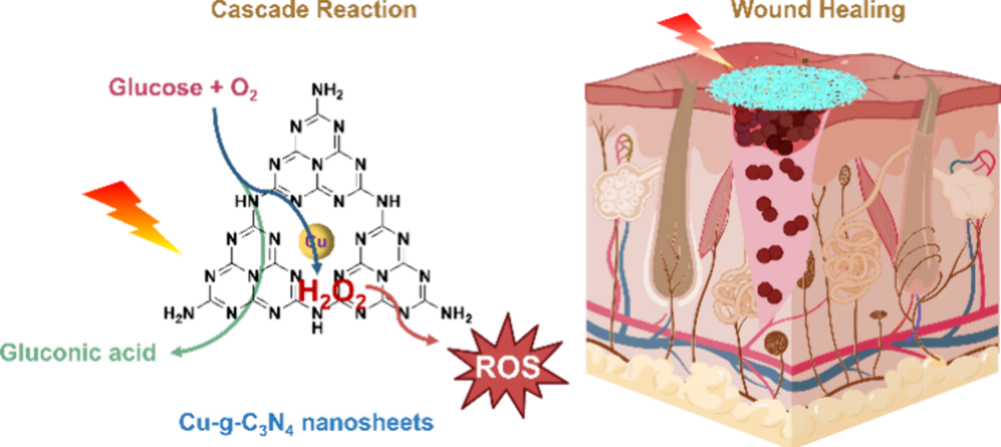

A very promising strategy to avoid bacterial drug resistance is to replace antibiotics with artificial nanozymes, but this has not yet been translated to the clinic. Here, we construct a single-atom nanozyme using graphitic carbon nitride nanosheets modified by copper (Cu-*g*-C_3_N_4_). This Cu-*g*-C_3_N_4_ nanosheet possesses both glucose oxidase-like and peroxidase-like activities responsible for reactive-oxygen-species generation by a cascade reaction to eradicate Gram-positive and Gram-negative multidrug-resistant bacteria. Cu-*g*-C_3_N_4_ is introduced into polycaprolactone (PCL) by electrospinning to obtain (Cu-*g*-C_3_N_4_/PCL) nanofibers, which can be used as a dressing for bacterially infected wounds. It is demonstrated that Cu-*g*-C_3_N_4_/PCL nanofiber dressings can eradicate bacterial infections and accelerate wound healing in a mouse model with a skin wound.

## Introduction

Antibiotic resistance represents an urgent threat to global public health.^1,2^ Bacteria tend to adhere to a surface such as damaged tissue or medical devices, which can cause chronic, persistent, or biomaterials-associated infections.^3,4^ Usually, these adhered bacteria produce polysaccharides, proteins, and eDNA, forming a matrix, in which they are encapsulated, known as a biofilm.^5−8^ Bacteria residing within a biofilm have many benefits, like shielding them from environmental stress, including antimicrobial challenges and the host immune system. Therefore, a much higher antibiotic dose is required to eradicate bacteria in biofilms than planktonic bacteria.^9^ Reactive oxygen species (ROS) can disintegrate the extracellular polymeric matrix of biofilms, disrupt the bacterial cell membrane, and damage DNA, leading to the death of Gram-positive and Gram-negative antibiotic-resistant bacteria.^10,11^ Glucose oxidase and horseradish peroxidase have been often employed in enzyme cascade catalysis to produce ROS from glucose and O_2_, respectively.^12,13^ However, natural enzymes, mostly proteins, may lose their catalytic activity in challenging environments such as the low pH inside biofilms.^14,15^ The isolation of natural enzymes is difficult and costly, which limits its application.^16^ To solve this problem, artificial nanozymes combining the unique properties of nanomaterials with the catalytic activities of natural enzymes need to be developed.^17,18^

Among various nanomaterials, two-dimensional (2D) nanosheets with a high surface area and defects for electron transport have been reported for their applications in photoinduction and photocatalysis to produce ROS.^19,20^ The electronic structure of graphitic carbon nitride (g-C_3_N_4_) with graphitic planes formed by triazine units can provide a visible-light response. Therewith, g-C_3_N_4_ can be used as a photosensitizer for the generation of ROS, which can be used as an antimicrobial. Previous studies have shown the potential of g-C_3_N_4_ as a bifunctional nonenzymatic cascade for glucose and peroxide detection.^21,22^ Nonetheless, limited by the low utilization efficiency of visible light, its therapeutic efficacy on bacterially infected wounds *in vivo* is not satisfying. Taking advantage of surface defects and topological insulation of nanosheets, which is enhancing electron transport, metal atoms have been added into these defects of the nanosheets to improve the enzymatic cascade efficiency.^21,23^

**Scheme 1:**
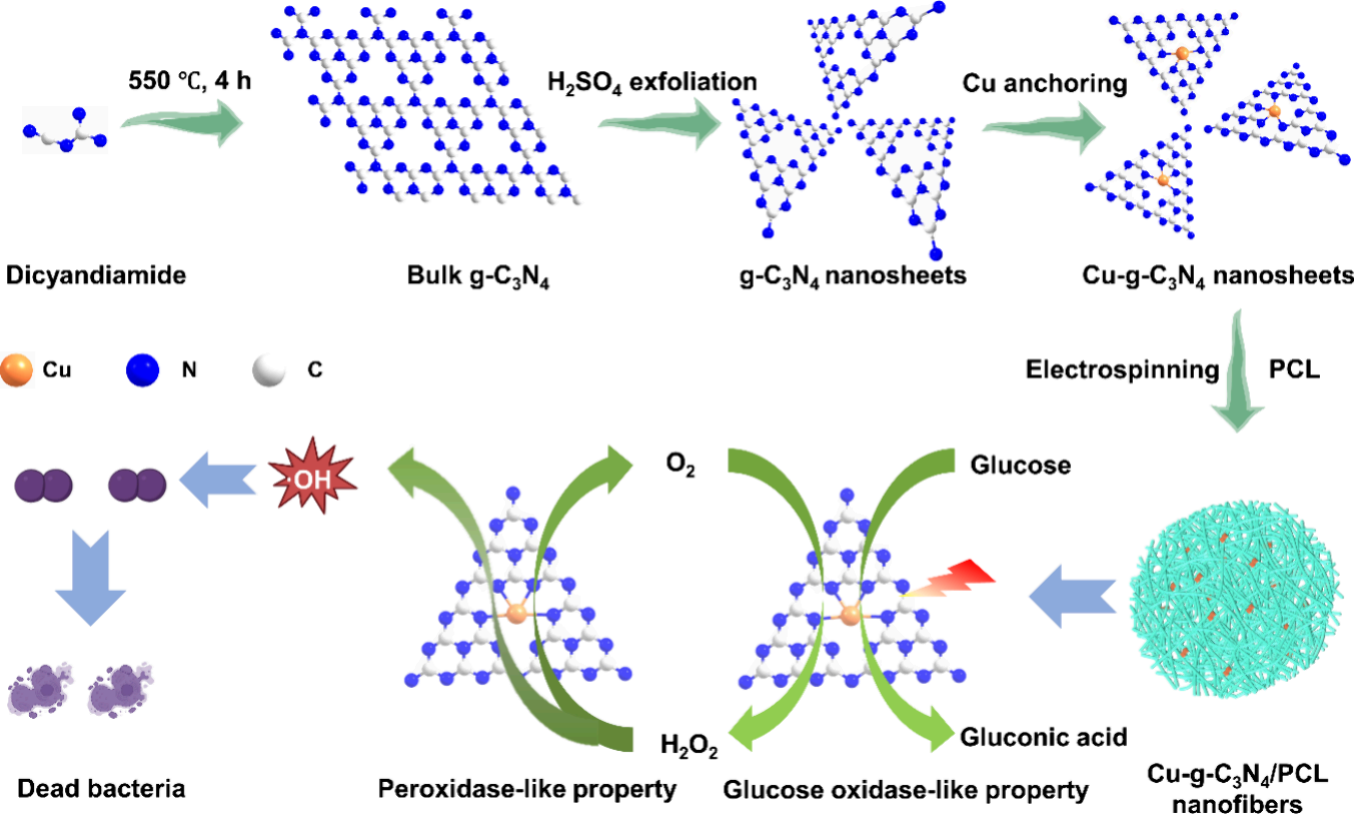
Synthesis of Single-Atom Cu-g-C_3_N_4_/PCL Nanofibers and the Reaction Mechanism of Cu-*g*-C_3_N_4_ Nanosheets as Glucose Oxidase-Like and Peroxidase-Like with Light Irradiation or Dark Conditions to Eradicate Bacteria The glucose oxidase-like Cu-*g*-C_3_N_4_ nanosheets oxidized glucose to generate gluconic acid and hydrogen peroxide (H_2_O_2_) in the presence of light and oxygen. The H_2_O_2_ generated *in situ* reacted with Cu-*g*-C_3_N_4_ as a peroxidase-like enzyme to format hydroxyl radicals (**·**OH) to kill bacteria.

Here, we present a bifunctional nanozyme, a single-atom copper-anchored g-C_3_N_4_ nanosheet (Cu-*g*-C_3_N_4_). Cu-*g*-C_3_N_4_ plays a dual role in glucose oxidase-like or peroxidase-like activity in chromogenic substrate oxidation with visible-light irradiation or dark conditions, respectively, to accomplish a cascade reaction (Scheme 1). H_2_O_2_ produced *in situ* from glucose oxidation and dioxygen reduction was utilized for peroxidation of a chromogenic substrate on Cu-*g*-C_3_N_4_ nanosheets to obtain hydroxyl radicals showing broad-spectrum activity against different multidrug-resistant bacteria. The excellent enzyme activity of Cu-*g*-C_3_N_4_ compared to that of g-C_3_N_4_ and other metals (Cr, K, Fe, and Zn) anchored with g-C_3_N_4_ arises from its high light absorption ability and surface adsorption. Furthermore, Cu-*g*-C_3_N_4_ nanosheets with polycaprolactone (PCL) nanofibers were prepared via electrospinning as a wound dressing to evaluate Cu-*g*-C_3_N_4_ performances on bacterial infection control. Cu-*g*-C_3_N_4_/PCL, as a dressing, was then systematically investigated for antibacterial and anti-inflammatory activities, biocompatibility, and wound healing potential *in vitro* and *in vivo*. This work uniquely integrates single-atom nanozymes on nanofibers as a dressing that can be used in hospitals during surgery and for the clothing of nurses and doctors, broadening the design and application of nanozymes.

## Results

### Synthesis and Characterizations of Cu-*g*-C_3_N_4_ Nanosheets

**Figure 1:**
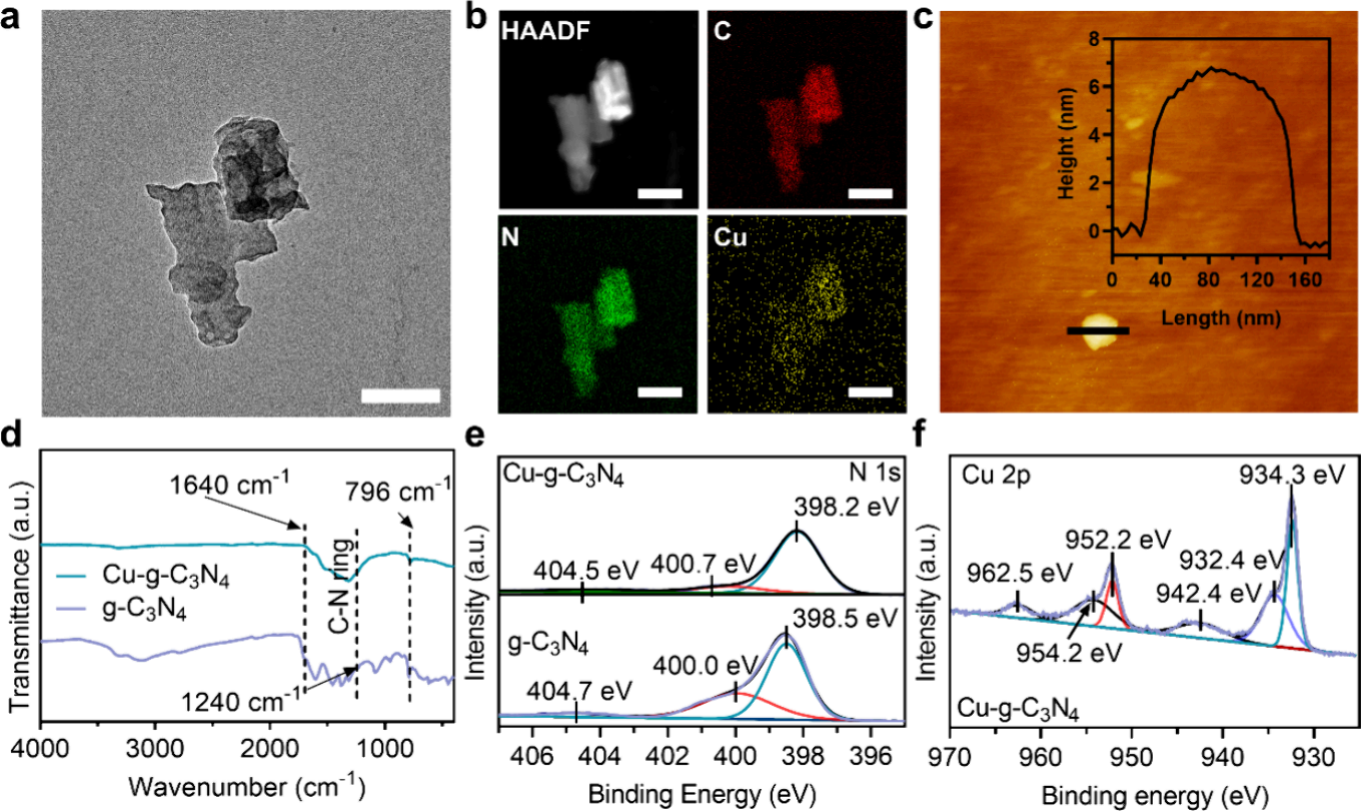
Structural characterizations of single-atom Cu-*g*-C_3_N_4_ nanosheets. (a) Transmission electron microscopy (TEM) image of Cu-*g*-C_3_N_4_ nanosheets. Scale bar = 100 nm. (b) High-angle annular dark-field (HAADF) of TEM images showing the distribution of C, N, and Cu. Scale bar = 100 nm. (c) Atomic force microscopy (AFM) image and a length and height profile of a Cu-*g*-C_3_N_4_ nanosheet. (d) Fourier transform infrared (FT-IR) spectra of g-C_3_N_4_ and Cu-*g*-C_3_N_4_ nanosheets. (e) X-ray photoelectron spectroscopic (XPS) narrow scan spectra of N 1s of g-C_3_N_4_ and Cu-*g*-C_3_N_4_ nanosheets. (f) XPS narrow scan Cu 2p from Cu-*g*-C_3_N_4_ nanosheets (Figure S4 shows a wide scan).

Density functional theory calculations revealed that Cu delivered the highest adsorption energy during adsorption to g-C_3_N_4_ compared to Fe, Cr, Zn, and K (Figure S1a). Cu-*g*-C_3_N_4_ had the highest light absorption (Figure S1b), and also Cu-*g*-C_3_N_4_ showed the highest antibacterial properties against *Staphylococcus aureus* Xen36 compared with Fe-*g*-C_3_N_4_, Cr-*g*-C_3_N_4_, K-*g*-C_3_N_4_, and Zn-*g*-C_3_N_4_ (Figure S1c,d). Therefore, Cu-*g*-C_3_N_4_ nanosheets were selected to investigate further. The synthesis and reaction mechanism of g-C_3_N_4_ and Cu-*g*-C_3_N_4_ nanosheets is illustrated in Scheme 1. Bulk g-C_3_N_4_ was synthesized and exfoliated by sulfuric acid to obtain g-C_3_N_4_ nanosheets (Figure S2). These nanosheets showed characteristic structures of g-C_3_N_4_ in X-ray powder diffraction spectra, with two characteristic diffraction peaks at 12.6 and 27.9°, corresponding to (100) and (002) planes,^24^ respectively (Figure S3). Introduction of Cu into g-C_3_N_4_ nanosheets resulted in a decrease in the crystallinity of the nanosheets. However, no peaks assigned to Cu hydroxides or oxides were detected (Figure S3), indicating that Cu atoms did not form crystalline phases in g-C_3_N_4_, which was confirmed by TEM where no crystalline lattices were observed on Cu-*g*-C_3_N_4_ nanosheets (Figure 1a). EDS elemental mapping illustrated the distributions of carbon, nitrogen, and copper (Figure 1b). The size of the Cu-*g*-C_3_N_4_ nanosheets was measured with AFM and was 120 nm in length and 6–7 nm in height (Figure 1c).

Quantification by inductively coupled plasma optical emission spectrometry (ICP-OES) detected that 16.3 wt % of Cu was successfully anchored in g-C_3_N_4_ nanosheets. FT-IR peaks at 3310 and 3140 cm^–1^ indicated the stretching vibrations of ν_O–H_ and ν_N–H,_ respectively;^25,26^ peaks at 1240–1640 cm^–1^ showed the presence of the C–N ring structure, and peaks at 796 cm^–1^ corresponded to the triazine structure of g-C_3_N_4_ (Figure 1d).^27^ The local framework composition of g-C_3_N_4_ like the C-NH_*x*_ bonding structure (404.5 eV), bridging nitrogen atoms in N–(C)_3_ (400.7 eV), and sp^2^-hybridized nitrogen atoms in C=N–C (398.2 eV) remained in Cu-*g*-C_3_N_4_ nanosheets (Figure 1e).^28^ Cu 2p XPS (Figure 1f) showed peaks at 934.3 and 954.2 eV, which are attributed to Cu 2p_3/2_ and Cu 2p_1/2_ of Cu^2+^,^29^ respectively. The peaks at 942.8 and 962.5 eV, due to the empty 3d shell of Cu^2+^,^30^ and the peaks at 932.4 and 952.2 eV are related to the presence of Cu 2p_3/2_ and Cu 2p_1/2_ of Cu^+^.^31^ In short, the structural skeleton of g-C_3_N_4_ was maintained, and Cu atoms were strongly integrated with g-C_3_N_4_ in the Cu-*g*-C_3_N_4_ nanosheets.

**Figure 2:**
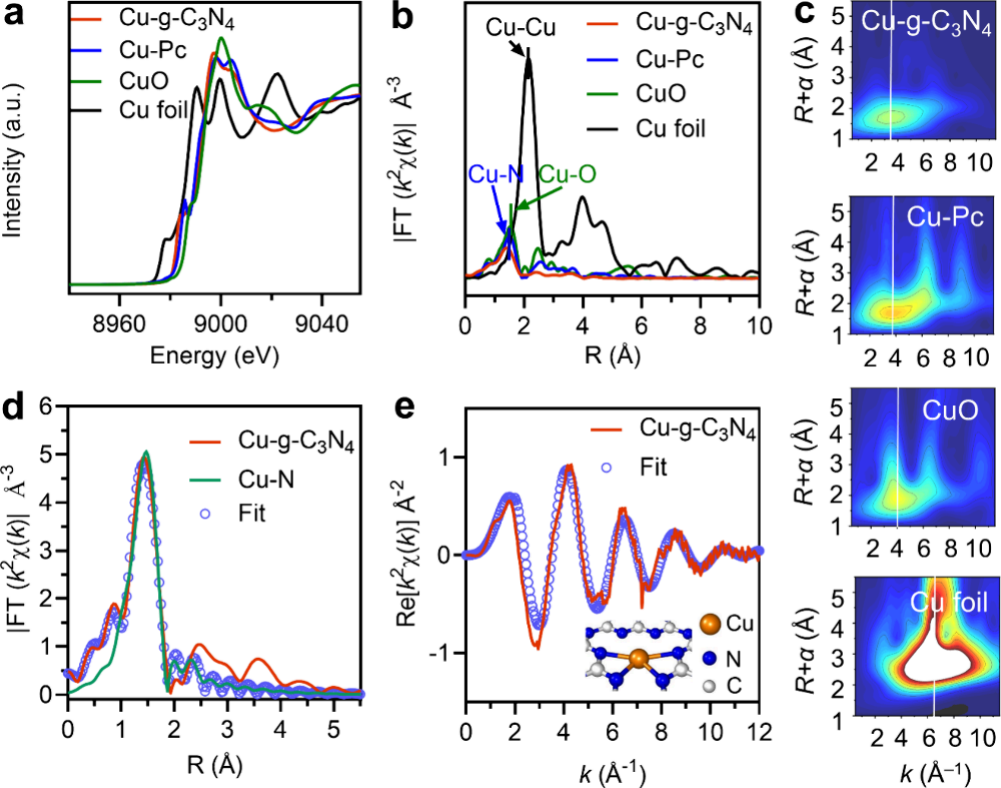
Atomic structural analysis of Cu-*g*-C_3_N_4_ nanosheets. (a) Cu K-edge XANES spectra of Cu-*g*-C_3_N_4_, copper phthalocyanine (Cu-Pc), CuO, and Cu foil. (b) *k*^3^-weighted Fourier-transformed (FT) spectra from extended X-ray absorption fine structure (EXAFS) of Cu-*g*-C_3_N_4_, Cu-Pc, CuO, and Cu foil. (c) Wavelet transform (WT) of the *k*^3^-weighted EXAFS for Cu-*g*-C_3_N_4_, Cu-Pc, CuO, and Cu foil. The white line is the *k*-value. (d) Fit of the Cu-*g*-C_3_N_4_ EXAFS spectrum in R space. (e) Fit of the Cu-*g*-C_3_N_4_ EXAFS spectrum in *k*-space; the inset shows the corresponding model structure.

To further investigate the binding of Cu in g-C_3_N_4_ nanosheets at the atomic level, X-ray absorption near-edge structure (XANES) and extended X-ray absorption fine structure (EXAFS) spectroscopy at the Cu K-edge were conducted. Cu foil, CuO, and Cu phthalocyanine (Cu-Pc) were chosen as reference materials for Cu–Cu, Cu–O, and Cu–N coordination materials, respectively. From Cu K-edge XANES spectra (Figure 2a), the absorption edge of Cu-*g*-C_3_N_4_ was positioned between the Cu foil and CuO, indicating that the oxidation valence state of the Cu atom was higher than that of metallic Cu^0^ and lower than that of Cu^2+^. The spectrum of Cu-*g*-C_3_N_4_ was very similar to Cu-Pc, indicating that the coordination of Cu atoms in Cu-*g*-C_3_N_4_ was Cu–N. For Fourier transform EXAFS spectra (Figure 2b), the curve of Cu-*g*-C_3_N_4_ nanosheets showed a clear peak at 1.4 Å, which is close to the single atomically dispersed Cu agents.^32^ No Cu–Cu coordination peak around 2.2 Å was observed in the Cu-*g*-C_3_N_4_ nanosheets, suggesting that all copper species exist as isolated single atoms. The wavelet transformation (Figure 2c) showed that the peak of Cu-*g*-C_3_N_4_ nanosheets tends to have a lower *k* value than that of CuO and Cu foil and is close to Cu-Pc, further confirming that Cu–Cu bonds were not present in Cu-*g*-C_3_N_4_ nanosheets and the Cu–N bond exists in Cu-*g*-C_3_N_4_ nanosheets. Quantitative EXAFS curve fitting analysis (Figure 2d,e and Table S1) was performed to investigate the structural parameters of Cu-*g*-C_3_N_4_. The Cu–N coordination number is ∼4.2, meaning that the isolated Cu atom is fourfold coordinated by N atoms (Figure 2e, inset). The calculated Cu–N_4_ mean bond distance in Cu-*g*-C_3_N_4_ is 1.92 Å. Summarizing, the Cu is single atomically anchored in g-C_3_N_4_ nanosheets to form single-atom Cu-*g*-C_3_N_4_ nanosheets.

### Glucose Oxidation and Peroxidase Cascade Catalytic Reactions

**Figure 3:**
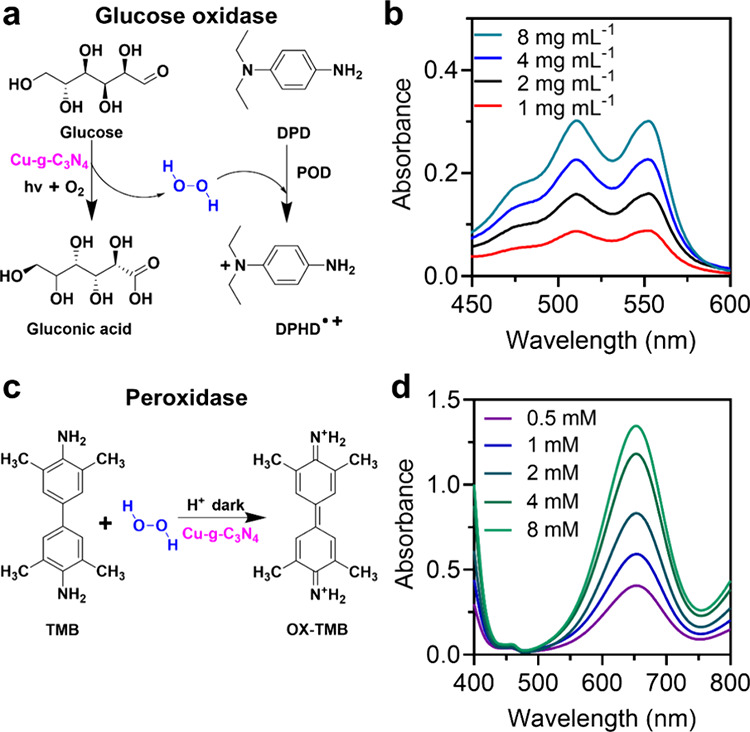
Glucose oxidase and peroxidase activities of single-atom Cu-*g*-C_3_N_4_ nanosheets. (a) Scheme of glucose oxidase-like reactions of Cu-*g*-C_3_N_4_ nanosheets. (b) UV–vis spectra of H_2_O_2_ generation from different glucose concentrations with Cu-*g*-C_3_N_4_ nanosheets (50 μg mL^–1^) in phosphate buffer (pH 7) and visible-light irradiation for 30 min (λ ≥ 420 nm, 0.2 W cm^–1^) using *N*,*N*-diethyl-l,4-phenylenediammonium sulfate (DPD) and peroxidase (POD). (c) Scheme of peroxidase-like reaction. (d) UV–vis spectra of Cu-*g*-C_3_N_4_ nanosheets for different H_2_O_2_ concentrations as the substrate in acetate buffer (0.1 M, pH 4) after 10 min incubation. The baseline in (b) and (d) have been corrected.

The mechanism of cascade enzymes’ sequential catalysis is illustrated in Scheme 1. The concentration of H_2_O_2_ was detected by the *N*,*N*-diethyl-1,4-phenylenediamine (DPD) and peroxidase by measuring the absorbance at 551 nm (Figure 3a). Cu-*g*-C_3_N_4_ nanosheets showed a higher glucose oxidase activity than g-C_3_N_4_ nanosheets (Figure S5), which is attributed to its increased light absorption capacity due to the Cu atom enhancing electron transportation (Figure S1a,b). The H_2_O_2_ concentration generated from glucose oxidation was calculated using eq 1:
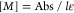
1in which [*M*] is the product (H_2_O_2_) concentration, Abs is the UV–vis absorbance of DPHD^•+^ at 551 nm, *l* = 1 cm, and ε = 21,000 M^–1^ cm^–1^.

The generation of H_2_O_2_ was 4.7 × 10^–6^, 8.1 × 10^–6^, 11 × 10^–6^, and 15 × 10^–6^ M from glucose concentrations of 1, 2, 4, and 8 mg mL^–1^, respectively, which shows that the yields were dependent on the glucose concentration (Figure 3b).

The intrinsic peroxidase-like activity of Cu-*g*-C_3_N_4_ nanosheets was determined by the oxidation of TMB (3,3′,5,5′-tetramethylbenzidine) (Figure 3c). OX-TMB generated from TMB and H_2_O_2_ was calculated using eq 1 with different parameters: [*M*] is the OX-TMB concentration generated from H_2_O_2_ and TMB, Abs is the UV–vis absorbance of OX-TMB at 652 nm, *l* = 1 cm, and ε = 39,000 M^–1^ cm^–1^. The OX-TMB concentrations were 1.0 × 10^–5^, 1.5 × 10^–5^, 2.1 × 10^–5^, 3.5 × 10^–5^, and 3.5 × 10^–5^ M from different H_2_O_2_ concentrations of 0.5, 1, 2, 4, and 8 mM, respectively. The yields of OX-TMB were dependent on the concentration of H_2_O_2_ (Figure 3d). The **·**OH generated from the cascade reaction was detected by terephthalic acid, which confirmed **·**OH generation after exposure to Cu-*g*-C_3_N_4_ (Figure S6). The above results show that the single-atom Cu-*g*-C_3_N_4_ nanosheet is an effective nanozyme to initialize the cascade reaction of glucose oxidation and H_2_O_2_ reduction.

### Density Functional Theory Calculations

**Figure 4:**
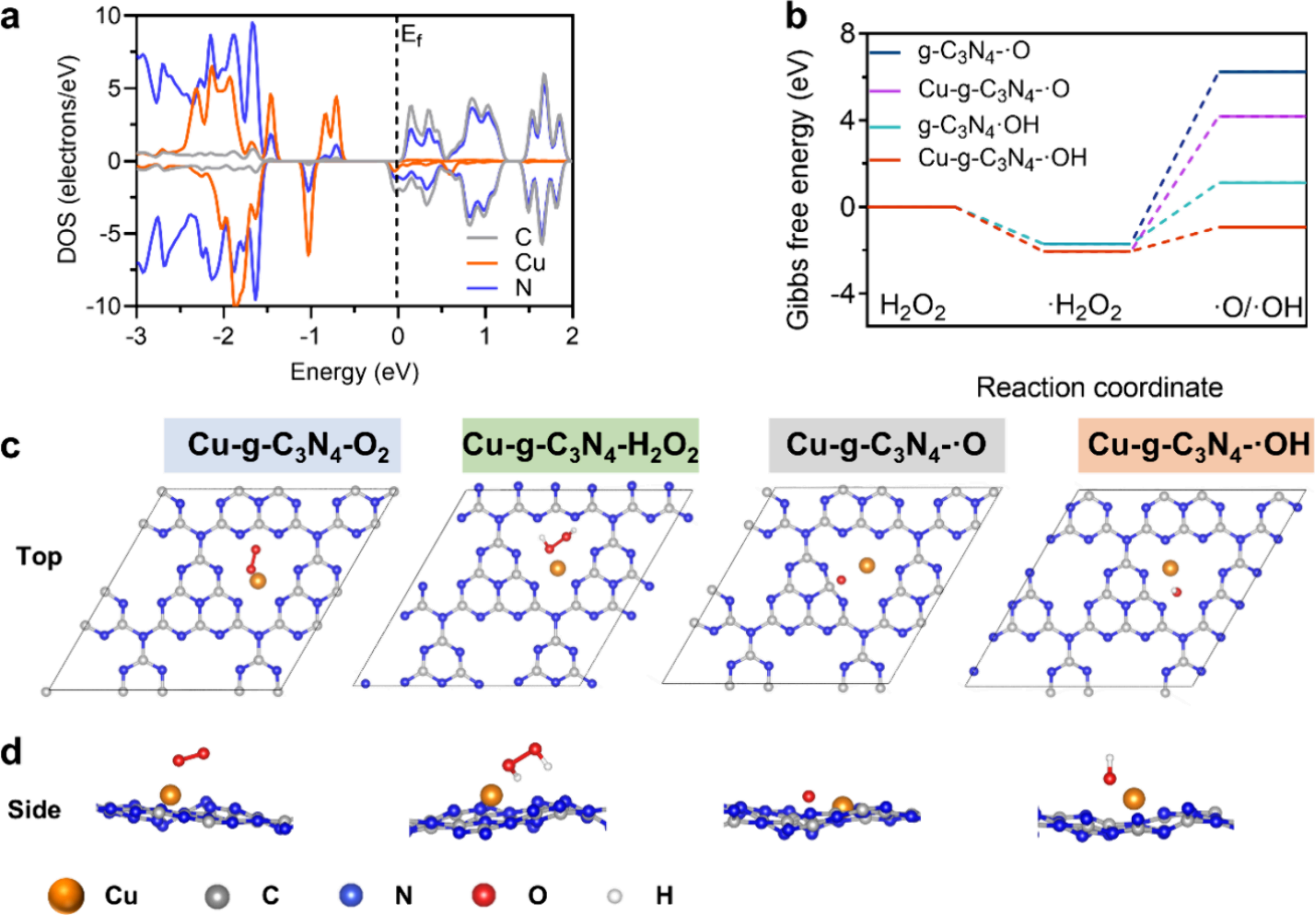
Density functional theory calculations on g-C_3_N_4_ and single-atom Cu-*g*-C_3_N_4_ nanosheets. (a) Density of electron states (DOS) and orbital distribution of the elements C, N, and Cu in Cu-*g*-C_3_N_4_ nanosheets; *E*_f_ is the Fermi level. (b) Gibbs free energy (Δ*G*) diagrams of **·**O or **·**OH on g-C_3_N_4_ and Cu-*g*-C_3_N_4_. Before the generation of **·**O and **·**OH, an intermediate **·**H_2_O_2_ is formed due to the decomposition of H_2_O_2_ on the nanosheet’s surface. The reaction energy is calculated from H_2_O_2_ to **·**H_2_O_2_, and then to **·**O or **·**OH. (c) Top view of the optimized structure of O_2_, H_2_O_2_, **·**O, and **·**OH on Cu-*g*-C_3_N_4_ nanosheets and (d) same as (c), which is the side view.

Strong binding of metal atoms on the substrate is mandatory for their catalytic activities and long-term uses.^33^ In Figure 4a, electron properties near the Fermi level (*E*_f_) obtained from Cu-*g*-C_3_N_4_ nanosheets were analyzed using the density of their energy states. A large overlap between N and Cu orbitals was observed, confirming a tight electron interaction between Cu and N, which is required for the strong anchoring of Cu single atoms on g-C_3_N_4_ nanosheets.

Strong adsorption of O_2_ and H_2_O_2_ on the surface of Cu-*g*-C_3_N_4_ nanosheets is crucial for the reaction efficiency (Scheme 1). The adsorption energies were calculated, and the corresponding structural configurations of each intermediate on g-C_3_N_4_ and Cu-*g*-C_3_N_4_ nanosheets showed different locations for the oxygen species in Figure 4c,d and Figure S7. The adsorption energy of O_2_ on Cu-*g*-C_3_N_4_ nanosheets (−2.29 eV) calculated using eq 2 was lower than on g-C_3_N_4_ nanosheets (0.40 eV), and also the adsorption energy of H_2_O_2_ onto Cu-*g*-C_3_N_4_ nanosheets (−2.77 eV) was lower than for g-C_3_N_4_ nanosheets (−2.34 eV). The lower adsorption energy caused a higher activity of glucose oxidase and peroxidase activity for the Cu-*g*-C_3_N_4_ nanosheets. For the formation of **·**OH from H_2_O_2_, the first step is the decomposition of H_2_O_2_ to form **·**H_2_O_2_, and the corresponding reaction energy is −2.05 eV for Cu-*g*-C_3_N_4_ and −1.77 for g-C_3_N_4_ nanosheets (Figure 4b and Table S2). The second step is the generation of **·**OH from **·**H_2_O_2_ by using g-C_3_N_4_ and Cu-*g*-C_3_N_4_ nanosheets. Decomposition of **·**H_2_O_2_ into water and the free oxygen radical (**·**H_2_O_2_ → H_2_O + **·**O) resulted in a reaction energy of 6.23 eV, higher than the energy for the generation of **·**OH (1.11 eV). Clearly, Cu-*g*-C_3_N_4_ showed a lower reaction energy for **·**H_2_O_2_ and **·**OH generation, making Cu-*g*-C_3_N_4_ nanosheets an efficient catalyst for H_2_O_2_ conversion.

### Antibacterial and Antibiofilm Activities

**Figure 5:**
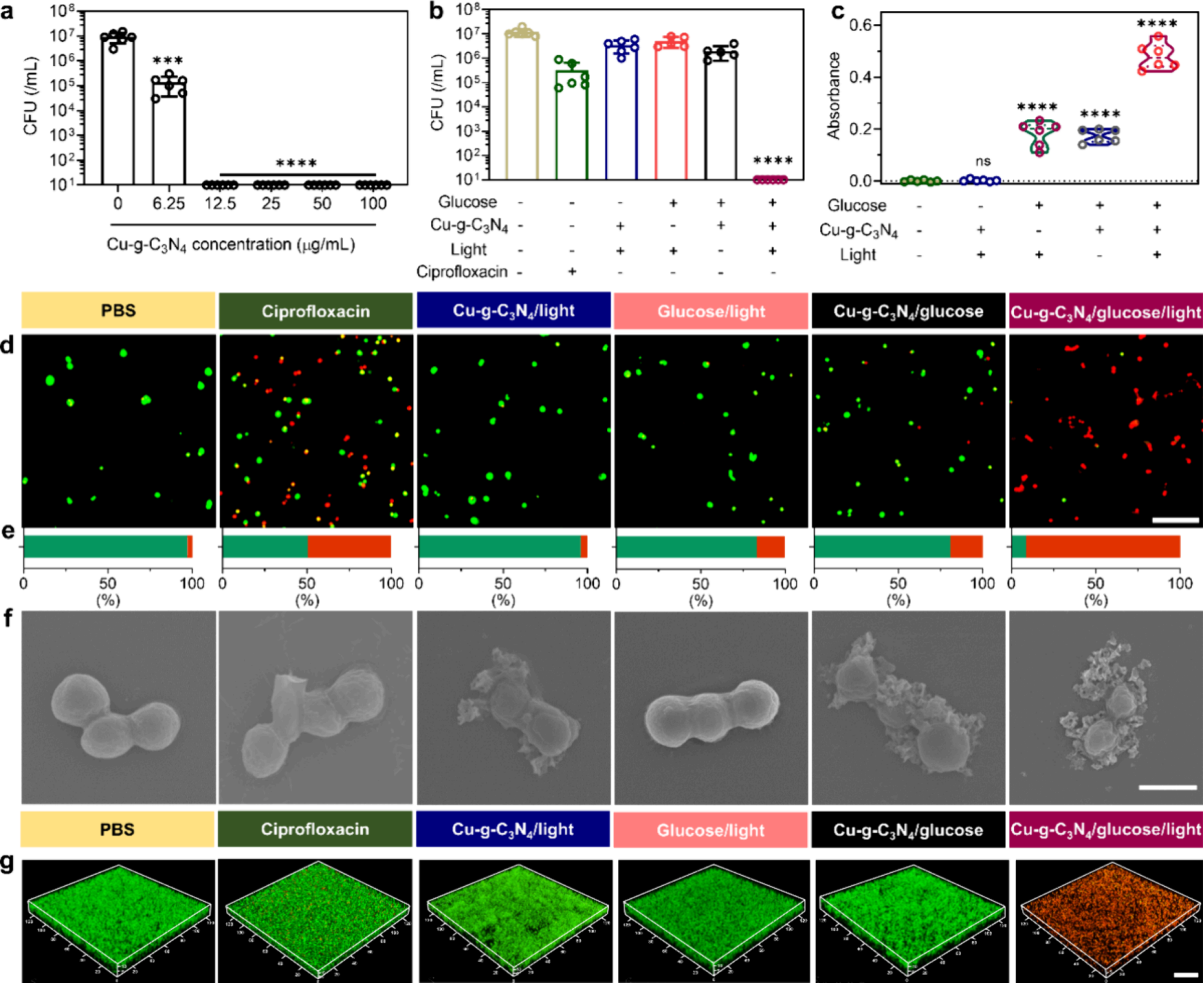
Killing efficacy of single-atom Cu-*g*-C_3_N_4_ nanosheets *in vitro* on planktonic *S. aureus* Xen36 and 24 h old biofilms exposed to Cu-*g*-C_3_N_4_ with or without glucose and light irradiation. (a) Killing efficacy on planktonic bacteria (10^7^ bacteria mL^–1^) of different concentrations of Cu-*g*-C_3_N_4_ nanosheets (glucose concentration: 4 mg mL^–1^, light irradiation: λ ≥ 420 nm, 0.2 W cm^–1^, 30 min). (b) Killing efficacy on planktonic bacteria (10^7^ bacteria m^–1^) of Cu-*g*-C_3_N_4_ nanosheets (12.5 μg mL^–1^) with or without the presence of glucose (4 mg mL^–1^) or light irradiation, PBS, and ciprofloxacin (12.5 μg mL^–1^) as controls. (c) Absorbance of the protein release after exposure to Cu-*g*-C_3_N_4_ with and without glucose and light irradiation of planktonic *S. aureus* Xen36 measured by using the BCA kit. (d, e) CLSM images and quantification of live/dead staining after 20 min exposure to PBS, ciprofloxacin (12.5 μg mL^–1^), and Cu-*g*-C_3_N_4_ nanosheets of planktonic *S. aureus* Xen36 (10^7^ bacteria mL; Cu-*g*-C_3_N_4_ nanosheets: 12.5 μg mL^–1^; glucose: 4 mg mL^–1^; light irradiation, scale bar is 10 μm. (f) FESEM images of planktonic *S. aureus* Xen36 after different exposures, scale bar is 1 μm. (g) CLSM images of 48 h old biofilms of *S. aureus* Xen36 after different exposures to Cu-*g*-C_3_N_4_ nanosheets and PBS and ciprofloxacin as controls and stained with live/dead stain; scale bar is 20 μm. Green fluorescent represents live bacteria stained by SYTO 9, and red represents membrane-damaged bacteria stained by propidium iodide. Statistical significance of differences with respect to PBS exposure (*n* = 6) indicates as ***p < 0.001 and ****p < 0.0001, and ns stands for no significant difference.

The antibacterial and antibiofilm activity of Cu-*g*-C_3_N_4_ nanosheets was evaluated *in vitro* for *S. aureus*, one of the most common bacteria causing wound infections (Figure 5). The optimal concentration of Cu-*g*-C_3_N_4_ nanosheets for killing all planktonic *S. aureus* Xen36 was 12.5 μg mL^–1^ (Figure 5a), which was used in all of the following experiments. Only Cu-*g*-C_3_N_4_ in the presence of glucose and light irradiation achieved antibacterial activity, which proves that the antibacterial activity of Cu-*g*-C_3_N_4_ nanosheets was due to the cascade reaction of glucose oxidation and H_2_O_2_ reduction (Figure 5b). This conclusion is supported by the CLSM micrographs shown in Figure 5d and Figure 5e, where 90% of bacteria were dead (cell membrane damaged) after exposure to Cu-*g*-C_3_N_4_/glucose/light. This was much higher than that of the bacteria exposed to Cu-*g*-C_3_N_4_ and glucose without light irradiation, where only 20% of bacteria were dead. Ciprofloxacin was used for comparison of the antibacterial activity of the Cu-*g*-C_3_N_4_/glucose/light treatment. The killing efficiency of ciprofloxacin against the drug-resistant *S. aureus* Xen36 was lower (50%) than the exposure to Cu-*g*-C_3_N_4_/glucose/light nanosheets. Field-emission scanning electron microscopy (FESEM) showed that Cu-*g*-C_3_N_4_ nanosheets aggregated around bacteria, leading to shrinkage of the bacteria when glucose and light were used (Figure 5f). Only bacteria exposed to Cu-*g*-C_3_N_4_/glucose/light demonstrated completely collapsed bacterial cell walls, which could be due to the ROS disruption of these cell walls. This result is consistent with the findings of the leakage of proteins from bacteria (Figure 5c), where Cu-*g*-C_3_N_4_/glucose/light treatment significantly increased the permeability of bacterial cell walls, leading to the leakage of bacterial cytoplasm (Figure 5c). Moreover, Cu-*g*-C_3_N_4_/glucose/light effectively damaged the cell walls of *S. aureus* Xen36 biofilms (Figure 5g), where only red staining was observed. The antibacterial activity of Cu-*g*-C_3_N_4_ was also evaluated against various multidrug-resistant bacteria, and broad-spectrum antibacterial activity was demonstrated regardless of Gram-positive or Gram-negative bacteria (Figure S8). Cytotoxicity results showed no toxicity to fibroblasts (L929 cells) after being incubated with Cu-*g*-C_3_N_4_ (12.5 μg mL^–1^) for 24 h (Figure S9). Summarizing, only Cu-*g*-C_3_N_4_ nanosheets irradiated with light in the presence of glucose achieved effective bacterial killing in their planktonic and biofilm modes of growth, indicating that Cu-*g*-C_3_N_4_ nanosheets are effective light-induced glucose oxidase-like and peroxidase-like enzymes involved in ROS generation.

### Synthesis of Cu-*g*-C_3_N_4_/PCL Nanofibers and Their Antibacterial Activity *In Vitro*

**Figure 6:**
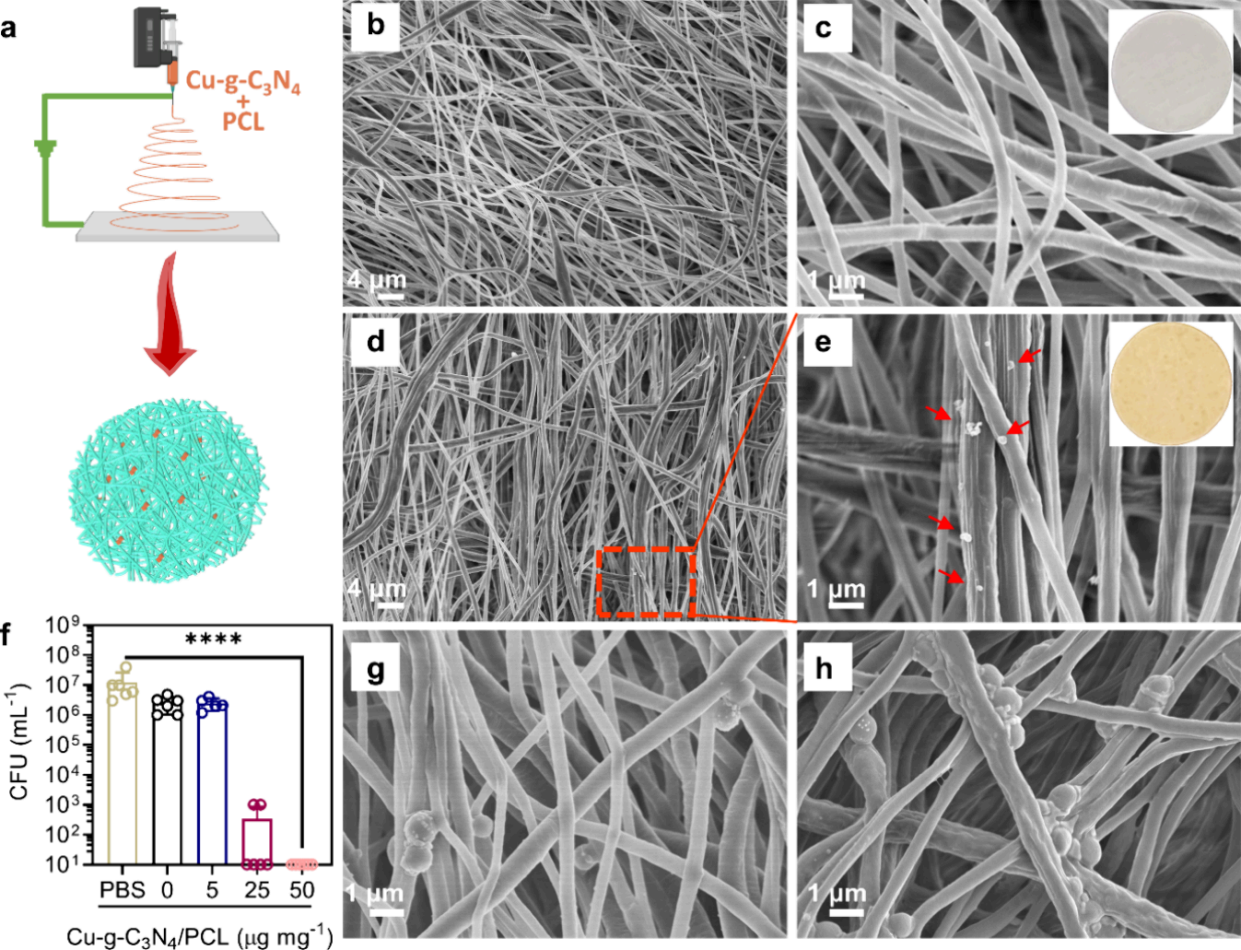
Synthesis of Cu-*g*-C_3_N_4_/PCL nanofibers and their antibacterial activity *in vitro*. (a) Schematic synthesis process of Cu-*g*-C_3_N_4_/PCL nanofibers. (b, c) FESEM of PCL nanofibers and inset in (c) is a piece of the PCL nanofiber. (d, e) FESEM of Cu-*g*-C_3_N_4_/PCL nanofibers and inset in (e) is a piece of Cu-*g*-C_3_N_4_/PCL nanofiber (red arrows are the Cu-*g*-C_3_N_4_ nanosheets). (f) Killing efficacy of Cu-*g*-C_3_N_4_/PCL nanofibers with different concentrations of Cu-*g*-C_3_N_4_ in the nanofibers in the presence of glucose and light irradiation of *S. aureus* Xen36 (2 × 10^7^ mL^–1^ in PBS, pH 7.4. Weight of nanofibers: 100 mg; glucose: 4 mg mL^–1^; light: λ ≥ 420 nm, 0.2 W cm^–1^, 30 min). (g) FESEM image of *S. aureus* Xen36 on PCL nanofibers and (h) the same as (g) but now on Cu-*g*-C_3_N_4_/PCL nanofibers with glucose and light for 30 min. Error bars indicate means ± SD (*n* = 6), ****p < 0.0001.

To apply Cu-*g*-C_3_N_4_ in a wound dressing, Cu-*g*-C_3_N_4_/PCL nanofibers were loaded with Cu-*g*-C_3_N_4_ onto PCL nanofibers with electrospinning technology (Figure 6a). PCL was used as the material for the nanofibers to prepare the wound dressing because it is a hydrophobic material that can be easily removed from the wound after treatment. In contrast to the smooth surface of pure PCL nanofibers (Figure 6b,c), Cu-*g*-C_3_N_4_/PCL nanofibers possessed a rough surface covered with Cu-*g*-C_3_N_4_/PCL (Figure 6d,e). The successfully synthesized Cu-*g*-C_3_N_4_/PCL nanofibers killed planktonic *S. aureus* Xen36 most effectively at a concentration of 50 μg mg^–1^ in the presence of glucose and light irradiation (Figure 6f). Bacterial morphology remains intact on PCL nanofibers (Figure 6g), but it is damaged on Cu-*g*-C_3_N_4_/PCL nanofibers (Figure 6h). The Cu-*g*-C_3_N_4_/PCL nanofibers showed zone of inhibitions for *S. aureus* Xen36 at a concentration of 25 μg mg^–1^ or higher (Figure S10). These findings provide evidence that Cu-*g*-C_3_N_4_/PCL nanofibers can be used as an antibacterial wound dressing *in vivo*.

### Cu-*g*-C_3_N_4_/PCL Nanofiber Wound Dressing for an Infectious Biofilm in a Skin Wound in a Mouse Model

**Figure 7:**
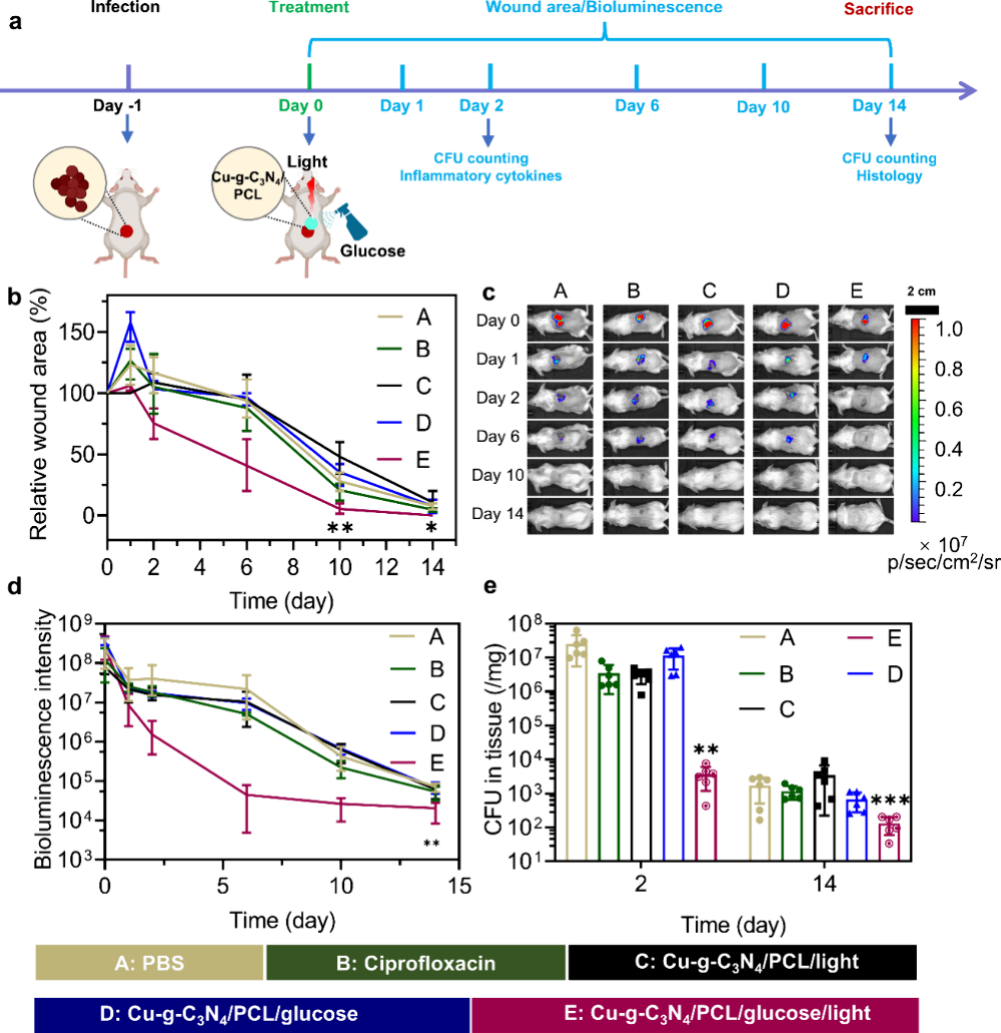
Cu-*g*-C_3_N_4_/PCL nanofibers as a wound dressing to treat *S. aureus* Xen36 biofilm infection *in vivo*. (a) Diagram of the animal experimental design of Cu-*g*-C_3_N_4_/PCL nanofibers as wound dressings for bacterial infection in the presence or absence of glucose (10 μL, 4 mg mL^–1^) and light irradiation for 30 min (λ ≥ 420 nm, 0.2 W cm^–1^). The PBS and ciprofloxacin (10 μL, 12.5 μg/mL) were used as the control groups. (b) Relative wound area with different treatments on days 0, 1, 2, 6, 10, and 14 calculated with ImageJ from Figure S11. (c) Bioluminescence images of mice with different treatments on days 0, 1, 2, 6, 10 and 14. (d) Bioluminescence intensity obtained from (c). (e) CFU counts of bacteria on days 2 and 14 in the wound tissue. Asterisks indicate statistically significant differences to the PBS treatment *p < 0.05, **p < 0.01, and ***p < 0.001.

Cu-*g*-C_3_N_4_/PCL nanofibers were prepared as wound dressings to treat bacterial infections in an open-wound infection model.^10^ The animal experimental design is illustrated in Figure 7a. From the images and quantification of wound areas (Figure 7b and Figure S11), wounds treated with Cu-*g*-C_3_N_4_/PCL/glucose/light cured faster than those treated with the other treatments. The bioluminescence intensity of *S. aureus* Xen36 bacteria decreased with time and disappeared after 2 days in mice treated with Cu-*g*-C_3_N_4_/PCL nanofibers in the presence of glucose and light (Figure 7c,d). Note that the low bioluminescence signal measured after day 2 is background bioluminescence (Figure 7d). CFU counts per milligram of tissue were significantly lower compared to PBS in wounds with Cu-*g*-C_3_N_4_/PCL/glucose/light treatment (Figure 7e). Treatment of the wound infected with *S. aureus* Xen36 with the antibiotic ciprofloxacin demonstrated a much slower wound healing than treatment with Cu-*g*-C_3_N_4_/PCL/glucose/light (Figure 7).

To evaluate the inflammatory process of the early phase on day 2 of wound healing, wound tissues were collected. Hematoxylin and eosin (H&E) and Masson tissue staining were performed on day 6. Wound sites treated with Cu-*g*-C_3_N_4_/PCL/glucose/light showed intact, multilayer epidermis with abundant, newly regenerated hair follicles and very few inflammatory cells (Figure S12a), as well as ample, orderly arranged collagen and vascular structures (Figure S12b), which indicates wound healing. On day 2, the expression of pro-inflammatory cytokines was measured. IL-1β, IL-6, and TNF-α decreased, and anti-inflammatory cytokine, IL-10, increased when the infected wound was exposed to Cu-*g*-C_3_N_4_/PCL/light/glucose nanofibers (Figure S12c–f). Histological analysis of mice’s major organs (heart, liver, spleen, lung, and kidney) after being treated with Cu-*g*-C_3_N_4_/PCL (Figure S13) for 14 days did not show abnormalities, which indicates that the Cu-*g*-C_3_N_4_/PCL nanofibers are biosafe and can be used as wound dressing material.

## Discussion

The emergence of catalytic nanozymes that emulate the functionalities of natural enzymes holds immense promise as potential agents for combating bacterial infections.^15,16^ Here, we first synthesized g-C_3_N_4_ nanosheets from bulk g-C_3_N_4_ and then introduced Cu to prepare a single-atom Cu-*g*-C_3_N_4_ nanozyme. The single-atom Cu-*g*-C_3_N_4_ nanozyme exhibits dual-glucose oxidase-like and peroxidase-like activities, orchestrating a cascade reaction that leads to the generation of targeted **·**OH (Scheme 1). In contrast to other cascade reaction devices utilizing extra commercial glucose oxidase,^34−37^ the Cu-*g*-C_3_N_4_ nanozyme possesses its own glucose oxidase-like activity, thereby reducing costs. On the other hand, directly injecting H_2_O_2_ into the human body is, in general, not desirable. In our study, the addition of H_2_O_2_ is not necessary since H_2_O_2_ is generated from naturally occurring glucose, which reduces the potential harm to the tissue. The Cu-*g*-C_3_N_4_ nanozyme showed to eradicate planktonic bacteria and biofilms in a low concentration (12.5 μg mL^–1^) with adequate biosafety (Figures S9 and S13), providing the potential of this nanozyme as a promising strategy for combating bacterial infections through innovative enzymatic mimicking.

A notable highlight of our study is the identification of Cu-*g*-C_3_N_4_ as a single-atom nanozyme (Figure 2). The promising attribution of single-atom nanozymes has been well recognized, offering a novel avenue for enhanced enzymatic catalysis.^38,39^ Our results provide evidence that Cu outperforms other metals when anchored with g-C_3_N_4_, emphasizing its superior efficacy in this context (Figure S1). Cu-*g*-C_3_N_4_, as a single-atom nanozyme, enhances the generation of **·**OH because of the strong adsorption of O_2_ and H_2_O_2_ molecules, which increases enzymatic catalysis. Carbon nitride nanozymes as bifunctional glucose oxidase-peroxidase have been used in solutions to generate ROS.^21,40,41^ In this study, we found that carbon nitride anchored with single-atom Cu can be used against planktonic multidrug-resistant bacteria (Figure S8) but also kill biofilm bacteria (Figure 5). By unveiling the potential of single-atom nanozymes such as Cu-*g*-C_3_N_4_, our work lays the foundation for a new frontier in the exploration of single-atom nanozymes within the enzymatic catalysis domain and broader biomedicine applications, offering promising avenues for transformative advancements in various scientific and practical arenas.

Cu-*g*-C_3_N_4_ nanosheets were introduced in the PCL nanofibers by electrospinning to obtain a wound dressing that can be applied on infected wounds (Figure 7). Although the control experiments showed that a wound is also healing due to the host defense system, Cu-*g*-C_3_N_4_/PCL nanofibers with the addition of glucose and light irradiation accelerate the healing rate by a factor of 5 even when compared to ciprofloxacin. Except for the eradication of bacteria, inflammatory cells migrate to the wound site to clear debris and pathogens (Figure S12). Subsequently, fibroblasts produce collagen, while angiogenesis occurs to establish new blood vessels, providing structural support and nourishment to the healing tissue.^42^ Pro-inflammatory factors, including cytokines such as IL-1β, IL-6, and TNF-α, orchestrate the immune response and stimulate tissue repair mechanisms. Concurrently, anti-inflammatory cytokines such as IL-10 help regulate inflammation and promote tissue remodeling. Both bacterial eradication and inflammatory response are required to accelerate infected wound healing.

Small quantities of electrospun nanofibers are necessary for biomedical and healthcare applications, which makes them very attractive for wound dressings, implants, drug delivery devices, and *in vitro* disease platforms.^43,44^ Therefore, if different functions could be developed and integrated into textiles, then Cu-*g*-C_3_N_4_/PCL nanofibers would increase opportunities to infiltrate them in human daily life. Nanofibers can be explored and utilized in the development of textiles for use in hospitals during surgery, as well as for the clothing of nurses and doctors.

## Study Limitations

Whereas this study successfully describes the antibacterial activity of single-atom Cu-*g*-C_3_N_4_/PCL/glucose/light, it is not investigated whether the concentration of glucose in the patient is enough or extra glucose needs to be added. The concentration of glucose for healthy human fasting blood is 0.6–0.99 mg mL^–1^, and that for diabetic patients is higher than 1.26 mg mL^–1^.^45,46^ For mice, the concentration range of glucose in blood is 0.7–1.2 mg mL^–1^, depending on strain and conditions.^47^ In our study, we added 4 mg mL^–1^ glucose when treating the wound, which is higher than available in humans and mice. Therefore, it is unclear whether it still works in patients without additional glucose using our material systems. Another challenge will be to reach the contact area between the wound and nanofibers with light irradiation. Probably a sheet instead of fibers prepared from Cu-*g*-C_3_N_4_/PCL will have better contact and can use the available glucose, but this needs further investigation. For broader applicability, it will be interesting to mix the nanofibers with textiles, but further investigation is also required on the ratio of the different fibers and the antibacterial activity.

## Conclusions

In conclusion, a novel nanozyme is introduced based on single-atom Cu-anchored g-C_3_N_4_ nanosheets with demonstrable bifunctional enzyme-mimicking activity of glucose oxidase and peroxidase. With the intrinsic glucose oxidase activity, the H_2_O_2_ generated from photoreaction of oxygen and glucose *in situ* triggers the subsequent peroxidase activity. This bifunctional Cu-*g*-C_3_N_4_ nanozyme showed broad-spectrum antibacterial activity against various multidrug-resistant bacteria in both planktonic and biofilm modes of growth. Subsequently, the Cu-*g*-C_3_N_4_ nanozyme is successfully incorporated in PCL fibers by electrospinning as a wound dressing material, which maintains the antibacterial capacity of Cu-*g*-C_3_N_4_ and serves as an ideal carrier of Cu-*g*-C_3_N_4_ nanozyme for *in vivo* applications. In the *in vivo* infection model, Cu-*g*-C_3_N_4_/PCL nanofibers in the presence of light irradiation and glucose promote faster wound healing, which is attributed to a synergy of antibacterial effects and inflammatory relief. It can be a promising wound dressing material to prevent bacterial infections and result in a faster recovery of the wound compared with the antibiotic ciprofloxacin.

## Experimental Section

### Chemicals and Materials

Dicyandiamide, sulfuric acid (H_2_SO_4_), Cu(NO_3_)_2_**·**H_2_O, Cr(NO_3_)_2_**·**9H_2_O, FeSO_4_**·**7H_2_O, Zn(NO_3_)_2_**·**6H_2_O, KCl, 3,3′,5,5′-tetramethylbenzidine (TMB), *N*,*N*-diethyl-1,4-phenylenediamine (DPD), sodium acetate, acetic acid, horseradish peroxidase, 4% paraformaldehyde fixation solution, glucose, terephthalic acid (TA), ciprofloxacin, and 5,5-dimethyl-1-pyrroline *N*-oxide (DMPO) were purchased from Macklin (Shanghai, China). H_2_O_2_ (30%) and absolute ethanol were obtained from Sigma-Aldrich (Shanghai, China). Sterile phosphate-buffered saline (PBS; 5 mM K_2_HPO_4_, 5 mM KH_2_PO_4_, and 150 mM NaCl, pH 7) was homemade and used in this study. Live/dead bacterial staining kit (SYTO 9, propidium iodide) was provided by Invitrogen (Shanghai, China). 0.25% trypsin/EDTA, agar powder (molecular weight, MW, 3000–9000), and BCA protein quantification kit were purchased from Solarbio (Beijing, China).

### Synthesis of Bulk g-C_3_N_4_, g-C_3_N_4_ Nanosheets, and Cu-*g*-C_3_N_4_ Nanosheets

A thermal polymerization method was used to prepare bulk g-C_3_N_4_, as previously described.^48^ Briefly, 6 g of dicyandiamide was added into a porcelain calcination boat and then heated from 20 to 550 °C within 4 h and kept at 550 °C for 4 h in air. After that, the bulk g-C_3_N_4_ were cooled to room temperature.

The g-C_3_N_4_ nanosheets were synthesized by dispersing 1 g of bulk g-C_3_N_4_ in 10 mL of H_2_SO_4_ (anhydrous) while stirring for 1 h. Subsequently, 10 mL of deionized water was dropped in the g-C_3_N_4_/H_2_SO_4_ solution until it turned from turbid to clear. The solution was added to 30 mL of absolute ethanol and stirred for 18 h, and the nanosheets were precipitated from the solution. Finally, the nanosheet suspension was dialyzed (membrane cutoff: 3500 kDa) in deionized water, which was refreshed every 3 h to remove both residual SO_4_^2–^ and ethanol until its pH was 7.0, determined by pH paper. The g-C_3_N_4_ nanosheets were harvested by centrifugation at 5000*g* for 5 min and dried at 60 °C for 18 h.

The Cu-*g*-C_3_N_4_ nanosheets were synthesized by adding 2.0 g of g-C_3_N_4_ nanosheets and 0.65 g of Cu(NO_3_)_2_**·**H_2_O into 10 mL of deionized water while stirring for 2 h. After drying on a hot plate (HP-05, Shanghai Xuesen Instrument Technology Co., Ltd.) for 8 h, the obtained powder was added in a semiclosed porcelain boat with a cover. It was heated in a tube furnace from room temperature to 130 °C with a heating rate of 10 °C min^–1^ under Ar flow and kept at this temperature for 9 h. After this step, the nanosheets were heated to 550 °C with a heating rate of 9 °C min^–1^ and kept at this temperature for 1 h. Then, the suspension with the Cu-*g*-C_3_N_4_ nanosheets was cooled down to room temperature, transferred to a 50 mL tube, washed five times with 30 mL of deionized water, and centrifuged at 5000*g* for 5 min. Finally, the sample was fully dried in a vacuum chamber at 60 °C for 18 h to obtain the powder of the nanosheets.

### Synthesis of Cu-*g*-C_3_N_4_/PCL Nanofibers for Wound Dressing

The Cu-*g*-C_3_N_4_/PCL nanofibers were prepared by electrospinning. First, dichloromethane (4 mL) and *N*,*N*-dimethylformamide (1 mL) were mixed by stirring for 10 min, and then 25 mg of Cu-*g*-C_3_N_4_ powder was dispersed in the mixed solution and continuously stirred for another 1.5 h. Then, 250 mg of PCL was added into the Cu-*g*-C_3_N_4_ suspension and mixed by shaking overnight. The solution was diluted by *N*,*N*-dimethylformamide containing 10% PCL to the following mass ratio of Cu-*g*-C_3_N_4_/PCL, 50, 25, and 5 μg mg^–1^. A 2 mL solution of each suspension was used for electrospinning. The parameters for electrospinning were electrical field strength 18 kV, flow rate of the solution 0.7 mL h^–1^, and DC voltage 18 kV. Electrospinning was stopped when the liquid was fully used, and the collected nanofibers were carefully cut with a scalpel. All of the nanofibers were placed in a vacuum at 25 °C for 24 h to remove the residual solvent. The morphologies of nanofibers were studied by field-emission scanning electron microscopy (FESEM, HITACHI, SU8010, Japan).

### Characterizations

X-ray powder diffraction (XRD, Bruker, D8 Advance, Germany) was employed to study the crystalline structure of g-C_3_N_4_ and Cu-*g*-C_3_N_4_ nanosheets with Cu Kα radiation at 40 kV and 40 mA. The morphologies and high-angle annular dark-field (HAADF) of as-prepared g-C_3_N_4_ bulk, g-C_3_N_4_ nanosheets, and Cu-*g*-C_3_N_4_ nanosheets were performed by transmission electron microscopy (TEM, FEI, F200S, USA). The functional groups were determined by a Fourier transform infrared spectrometer (Bruker, TENSOR II, Germany). The nanosheets were mixed with KBr and pressed in a tablet, and a blank KBr tablet was used as the background. Scans were recorded over the wavenumber range from 400 to 4000 cm^–1^ with a resolution of 0.4 cm^–1^, and 16 scans were made and averaged. The height of the samples was measured by an atomic force microscope (AFM, Bruker, WM2017014, USA) with a silicon tip (height 8–20 μm, opening angle of ca. 30–40°, apex radius 10 nm). A UV–vis spectrophotometer (UV-2700, Agilent, Cary 5000, USA) was used at a wavelength range of 320–800 nm for determining the light absorption ability of g-C_3_N_4_, Cr-*g*-C_3_N_4_, Zn-*g*-C_3_N_4_, Fe-*g*-C_3_N_4_, K-*g*-C_3_N_4_, and Cu-*g*-C_3_N_4_ nanosheets. X-ray photoelectron spectroscopy (XPS, Kratos Analytical Ltd., Axis Ultra DLD, Germany) was used to analyze the elemental surface composition. X-ray production was from Al-coated anodes using a spot size of 400 μm. Wide scans were over the binding energy range of 200–1100 eV with a pass energy of 150 eV. High-resolution narrow scans were made at a pass energy of 50 eV, including N_1s_ (395–407 eV) and Cu_2p_ (925–970 eV) spectral regions. The amount of Cu in Cu-*g*-C_3_N_4_ was measured by an inductively coupled plasma optical emission spectrometer (ICP-OES, Varian, 700-ES, USA).

### X-ray Absorption Near-Edge Structure (XANES) Analysis

To determine the bond of Cu in g-C_3_N_4_ nanosheets, Cu K-edge analysis was done with Si (111) crystal monochromators at the BL11B beamlines at the Shanghai Synchrotron Radiation Facility (SSRF) (Shanghai, China). Powder was pressed into thin sheets of 1 cm diameter and sealed using Kapton tape. The X-ray absorption fine structure (XAFS) spectra were recorded at room temperature using a four-channel silicon drift detector (SDD) Bruker 5040. Cu K-edge extended X-ray absorption fine structure (EXAFS) spectra were recorded in transmission mode and analyzed by the software codes Athena and Artemis. The spectra were normalized to 1, and the background was subtracted using the Athena program from the software package (IFFEFIT). The *k*^3^-weighted χ(*k*) data in the *k*-space ranging from 3 to 13 Å^–1^ (multiplied by a Hanning window function with d*k* = 1.0 Å^–1^) were Fourier transformed to obtain radial distribution functions (R space). In order to determine the detailed structural parameters around Cu atoms in the samples, quantitative curve-fittings were carried out for the Fourier transformed *k*^3^χ(*k*) in the R space between 1.2 and 3 Å using the Artemis module of IFEFFIT. Effective backscattering amplitudes *F*(*k*) and phase shifts Φ(*k*) of all fitting paths were calculated by the ab initio code FEFF 6.0. During the fitting of the Cu-*g*-C_3_N_4_ powder, the amplitude reduction factor *S*_0_^2^ was fixed to the best-fit value of 1.00, and the Cu–N coordination number was fixed to 2.0 while the interatomic distance (*R*), the Debye–Waller factor (σ^2^), and energy shift (δ*E*_0_) were allowed to vary. The fit quality was then evaluated using the *R*-factor and reduced Chi-square. The wavelet-transformed EXAFS spectra were obtained using the WTEXAFS program.

### Glucose Oxidase-Like Activity

Glucose oxidase-like activities of g-C_3_N_4_ and Cu-*g*-C_3_N_4_ were determined by measuring the generation of hydrogen peroxide using *N*,*N*-diethyl-l,4-phenylenediammonium sulfate (DPD) and horseradish peroxidase. First, 100 mg of DPD was dissolved in 10 mL of 0.1 M H_2_SO_4_ and stored in the dark at 5 °C, for a maximum of 1 week. 10 mg of horseradish peroxidase from horseradish was dissolved in 10 mL of distilled water and stored at 5 °C, for a maximum of 1 week. The glucose oxidation was performed in a quartz cuvette (5 mL) containing different concentrations (1, 2, 4, and 8 mg mL^–1^) of glucose and 0.2 mg of Cu-*g*-C_3_N_4_ in 4 mL of PBS (pH 7.0). The photoreaction assay was done in air with irradiation for 10 min (λ ≥ 420 nm) to generate H_2_O_2_, and the solution was filtered to remove the nanosheets. The CEL-HXF300-T3 xenon lamp (Ceaulight, Beijing, China) was used as the excitation light source to investigate the oxidation of glucose, and the distance between sample and lamp was set to 12 cm, with a 0.2 W cm^–2^ and 420–780 nm irradiation wavelength range. The concentration of generated H_2_O_2_ was determined by the DPD colorimetric method, as shown in Figure 3a,b. Briefly, 1 mL aliquots containing generated H_2_O_2_ were mixed with 3 mL of 10 mM phosphate buffer (pH 6), and then 50 μL of DPD solution (10 mg mL^–1^) and 50 μL horseradish peroxidase (1 mg mL^–1^) were added to the solution and mixed with pipetting and immediately measured using an UV–vis spectrophotometer. The absorbance at 551 nm was measured to determine the concentration of DPD^•+^ and used for calculating the H_2_O_2_ generated.^49^

### Peroxidase-Like Activity

For the peroxidation reaction, 800 μL of H_2_O_2_ (0.1, 0.5, 1, 2, 4, and 8 mM) was added to 3.9 mL of acetate buffer (0.1 M, pH 4.2), 200 μL of TMB (12 mM), and 100 μL of Cu-*g*-C_3_N_4_ (2.5 mg mL^–1^ in PBS, pH 7.4). The mixture was incubated in the dark for 10 min at room temperature and filtered to remove the nanosheets. The absorbance of the solution was measured at 652 nm, and the concentration of the oxidized product of TMB with ε = 39,000 M^–1^ cm^–1^.^21^

### Computational Methods

Theoretical calculations of Cu on g-C_3_N_4_ nanosheets were carried out with a computational quantum mechanical modeling using the density functional theory to investigate the electronic structure using the Vienna Ab initio Simulation Package (VASP 5.4.4). The projected augmented wave (PAW) method was used to describe the interactions of electrons and ions. The Perdew–Burke–Ernzerhof (PBE) approximation of generalized gradient approximation (GGA) was employed to obtain the exchange and correlation energy. The plane-wave expansion of the Kohn–Sham orbitals was expanded to a kinetic energy cutoff of 450 eV. The convergence criterion of 10^–4^ eV was set for electronic self-consistency, and structural optimizations were processed until the force on each atom was less than 0.02 eV Å^–1^. The parameters of the unit cell were *a* = *b* = 14.27 Å, *c* = 20 Å, α = β = 90°, and γ = 120°. The 20 Å vacuum layer was added to the surface to eliminate the artificial interactions between periodic images. The weak interaction was described by the DFT+D3 method using empirical correction in Grimme’s scheme.^50^

The adsorption energy (*E*_a_) was calculated with the following equation:
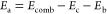
2where *E*_comb_ stands for the total energy of each metal adsorbed on the nanosheet (-*g*-C_3_N_4)_, *E*_c_ represents the energy of g-C_3_N_4_, and *E*_b_ is the energy of each metal.

The Gibbs free energies were calculated from the following equation:
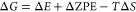
3where Δ*E* is the adsorption energy of **·**H_2_O_2_, **·**OH, and **·**O on the Cu-*g*-C_3_N_4_ nanosheet; ΔZPE and Δ*S* are the zero-point energy change and entropy change of **·**H_2_O_2_, **·**OH, and **·**O, respectively. *T* is the temperature (298.15 K).

From density functional theory, adsorption energies can be calculated (eq 2). In this work, adsorption energy refers to the energy necessary for atoms (Fe, Cr, Zn, K, and Cu) to adsorb onto g-C_3_N_4_ nanosheets and molecules (O_2_ and H_2_O_2_) to adsorb onto g-C_3_N_4_ and Cu-*g*-C_3_N_4_ nanosheets. A lower adsorption energy indicates that it is easier for the atoms to adsorb onto g-C_3_N_4_ nanosheets and molecules to adsorb onto Cu-*g*-C_3_N_4_. The Gibbs free energy (eq 3) is the thermodynamic potential that is minimized when a system reaches chemical equilibrium. Reaction energy is obtained by calculating the Gibbs free energy change between **·**O/**·**OH and **·**H_2_O_2_ on g-C_3_N_4_ or Cu-*g*-C_3_N_4_, and the lower the reaction energy, the easier it is for the reaction to occur.

### Bacterial Culturing, Growth Condition, and Harvesting

*Staphylococcus aureus* Xen36 (PerkinElmer, Inc., Waltham, Massachusetts) was grown on tryptone soy agar (TSA, Oxoid, Basingstoke, UK) with 200 μg mL^–1^ kanamycin at 37 °C under aerobic conditions. A single colony was added to 10 mL of tryptone soy broth (TSB) and incubated in humidified air at 37 °C for 24 h and used to inoculate (1:20) main culture (200 mL) and grown for 16 h. Bacterial cultures were harvested by centrifugation for 5 min at 5000*g* and washed twice with PBS (pH 7.4). Bacteria were suspended in PBS to the concentrations required for the experiments as determined by a Bürker-Türk counting chamber.

The multidrug-resistant ESKAPE strains (*Acinetobacter baumannii* 2349, *S. aureus* 6114, *Enterobacter faecium* 1762, *Klebsiella pneumoniae* 6696, *Pseudomonas aeruginosa* 3086, *Enterobacter* 3737) were isolated from patients from the First Affiliated Hospital of Wenzhou Medical University, Wenzhou, China. In the hospital, the ESKAPE strains were tested for multidrug resistance.^51,52^ The Gram-positive bacteria were cultured in TSB, and Gram-negative bacteria were cultured in LB. Culturing and harvesting were performed as described above.

For biofilm growth condition, 800 μL of *S. aureus* Xen36 suspension (10^8^ bacteria mL^–1^) was added into a glass-bottom cell culture dish (Φ14 mm) and incubated in humidified air at 37 °C for 1 h to allow the bacteria to adhere to the surface. Next, bacterial suspensions were discarded and washed with 800 μL of PBS to remove unattached bacteria, and 800 μL of TSB was added to each well and incubated at 37 °C. After 24 h, the culture medium was refreshed and cultured for another 24 h to form biofilms.

### Evaluation of Antibacterial Performance with Glucose Oxidase-Like Activity

100 μL of *S. aureus* Xen36 suspension (2 × 10^7^ bacteria mL^–1^) in PBS was added to a 96-well plate. Then, 10 μL of glucose (8 mg mL^–1^) and 90 μL of nanosheet suspension 0, 6.3, 12.5, 25, 50, and 100 μg mL^–1^ in PBS were added to the bacterial suspension. The plate was kept at room temperature with or without irradiation by a xenon lamp for 30 min (λ ≥ 420 nm, 0.2 W cm^–2^). Then, the bacterial suspension was serially diluted with PBS (pH 7.4), and 10 μL droplets were spotted onto TSB agar plates and incubated in humidified air at 37 °C overnight. CFUs were counted on the agar plates. For the killing efficacy on planktonic bacteria, bacterial suspensions were exposed to PBS, ciprofloxacin (12.5 μg mL^–1^), Cu-*g*-C_3_N_4_/light, glucose/light, Cu-*g*-C_3_N_4_/glucose, and Cu-*g*-C_3_N_4_/glucose/light for 30 min, the bacteria were spread on an agar plate, and CFUs were enumerated as described above. In addition, the antibacterial performance of g-C_3_N_4_, Cr-*g*-C_3_N_4_, Zn-*g*-C_3_N_4_, Fe-*g*-C_3_N_4_, and K-*g*-C_3_N_4_ nanosheets was tested as described above (Figure S1).

Antibacterial performance of Cu-*g*-C_3_N_4_ was also determined for the ESKAPE panel pathogens. Gram-positive bacteria were cultured in TSB medium, and Gram-negative bacteria were cultured in LB medium (Figure S8).

Protein leakage of the bacteria in the supernatant after exposure to Cu-*g*-C_3_N_4_ nanosheets was determined using the BCA protein detection kit and followed a previously reported method.^53^ Briefly, bacterial suspension exposed to PBS, Cu-*g*-C_3_N_4_/light, glucose/light, Cu-*g*-C_3_N_4_/glucose, and Cu-*g*-C_3_N_4_/glucose/light for 30 min was centrifuged at 5000*g* for 10 min at 4 °C and the protein concentration was determined in the supernatant. Bacteria in PBS without glucose or without light irradiation served as the controls.

FESEM (HITACHI, SU8010, Japan) was used to visualize the interaction of bacteria with the Cu-*g*-C_3_N_4_ nanosheets. *S. aureus* Xen36 suspensions were exposed to Cu-*g*-C_3_N_4_, as described above. The exposed bacterial suspension was added onto a silicon wafer (L × W × H, 5 mm × 5 mm × 0.45 mm) in a 48-well plate for 2 h to allow bacterial adhesion. Then, the bacteria were fixed with 4% paraformaldehyde fixative solution (Macklin, Beijing, China) for 40 min and dehydrated with ethanol solutions with increasing concentrations (30, 50, 70, 90, and 100%) for 20 min successively. The silicon wafer was dried at 4 °C, sprayed with gold, and analyzed on a field-emission scanning electron microscope.

The viability of *S. aureus* Xen36 after exposure to Cu-*g*-C_3_N_4_ nanosheets and ciprofloxacin and PBS as controls, as described above, was evaluated with a Live/Dead BacLight bacterial viability kit. Briefly, the bacteria were stained with SYTO 9 and propidium iodide (PI) for 20 min and analyzed with a Nikon A1 confocal laser scanning microscope (Nikon, A1, Japan). SYTO 9 (green fluorescent determining live bacteria) and PI (red fluorescent determining cell wall damaged bacteria) were excited by the 488 and 561 nm laser, respectively, and their emissions were collected at 500–550 and 570–620 nm. The percentage of SYTO 9-stained and PI-stained bacteria in confocal images was calculated by ImageJ (National Institutes of Health, USA).

### Evaluation of Biofilm Eradication Using Cu-*g*-C_3_N_4_ Nanosheets

*S. aureus* Xen36 biofilms grown on a glass-bottom cell culture dish for 48 h as described above, were exposed to 1 mL Cu-*g*-C_3_N_4_ nanosheets (12.5 μg mL^–1^) and glucose (4 mg mL^–1^) in PBS. The dish was kept at room temperature with or without irradiation by a xenon lamp for 30 min (λ ≥ 420 nm, 0.2 W cm^–2^). As controls, *S. aureus* Xen36 biofilms were exposed for 30 min in the dark to 1 mL of ciprofloxacin (12.5 μg mL^–1^) and PBS. Biofilms were stained with SYTO 9/PI for 30 min as described above and then imaged with confocal laser scanning microscopy.

### Evaluation of Antibacterial Properties of Cu-*g*-C_3_N_4_/PCL Nanofibers *In Vitro*

Cu-*g*-C_3_N_4_/PCL (1 mg, 10 mm diameter, 1 mm thickness) at different concentrations were added to 190 μL of *S. aureus* Xen36 (10^7^ mL^–1^) in PBS in a 48-well plate. 10 μL of glucose (80 mg mL^–1^) was then added into each well at the final concentration of 4 mg mL^–1^. The plate was illuminated by a light source for 30 min (λ ≥ 420 nm, 0.2 W cm^–2^); PBS without nanofibers were used as controls. The resultant bacterial suspension was serially diluted with PBS, and 10 μL droplets were spotted onto TSB agar plates and incubated at 37 °C overnight. CFUs were enumerated on the agar plates.

### Infected Murine Wound Model and Evaluation of Wound Healing with Cu-*g*-C_3_N_4_/PCL Dressings

Forty ICR female mice (6–8 weeks, 20–30 g) were purchased from Zhejiang Vital River Laboratory Animal Technology Co., Ltd., and housed in a specific-pathogen-free room. The animal experimental protocols were reviewed and approved by the Institutional Animal Care and User Committee, Wenzhou Institute, University of Chinese Academy of Sciences. The hind part of ICR female mice was shaved, and a circular, full-thickness skin wound with a diameter of 6 mm was made by a sterilized puncher (TEN-WIN). The wounds were then inoculated with *S. aureus* Xen36 (10 μL, 10^11^ bacteria mL^–1^) in PBS and randomly divided into four groups (eight mice per group). After 24 h (day 0), mice were anesthetized, and the wounds were treated with PBS (control), ciprofloxacin (10 μL, 12.5 μg mL^–1^), Cu-*g*-C_3_N_4_/PCL/light (5 mm-diameter Cu-*g*-C_3_N_4_/PCL with 10 min light irradiation (λ ≥ 420 nm, 0.2 W cm^–2^)), Cu-*g*-C_3_N_4_/PCL/glucose (5 mm-diameter Cu-*g*-C_3_N_4_/PCL, 4 mg mL^–1^ glucose in the dark), and Cu-*g*-C_3_N_4_/PCL/glucose/light (5 mm-diameter Cu-*g*-C_3_N_4_/PCL, 4 mg mL^–1^ glucose with l0 min light irradiation (λ ≥ 420 nm, 0.2 W cm^–2^)). At different time intervals (day 0, 1, 2, 6, 10, and 14), the bioluminescent images of the wounds were recorded by the IVIS Lumina XRMS Series III bio-optical imaging system (PerkinElmer, exposure time: 30 s, binning factor: 4, 1 F/Stop, open emission filter). Regions of interest were manually created for each image, and bioluminescence intensity was calculated. The wound sizes of the mice were recorded on days 0, 1, 2, 6, 10, and 14, and the wound area was measured and calculated by ImageJ (National Institutes of Health, USA).

In a parallel study, on day 2 post-treatment, three mice in each group were sacrificed, and the wound tissues were collected and immersed in PBS. The tissues were homogenized by using an ultrasonic cell pulverizer (HN150-Y, Hannuo). Meanwhile, inflammatory factors (IL-1β, IL-6, TNF-α, and IL-10) in wound tissue were determined using enzyme-linked immunosorbent assay (ELISA kit) following the manufacturer’s instructions, and the optical density (OD) value at 450 nm was measured with a microplate reader (Epoch2). The homogenized tissues were serially diluted with PBS and 10 μL droplets were spotted on TSB agar plates and cultured in humidified air at 37 °C overnight. CFUs were enumerated on the agar plates.

On day 14 post-treatment, all mice were sacrificed. Wound tissue and organs (heart, liver, spleen, lung, kidney) of the mice were fixed in 10% formalin, decalcified (wound tissue), embedded in paraffin, and sectioned in slices with a thickness of 5 μm; the slices were stained with hematoxylin and eosin (H&E) and Masson staining as previously published.^54^ The hematoxylin and eosin stain the nucleus (blue) and cytoplasm (red) to observe inflammatory cells. Masson staining shows a clear view of collagen fibers deposition and reorganization (blue).

### Statistical Analysis

All experiments were carried out in triplicate with separately cultured bacteria or cells. Results were expressed as means ± standard deviations (SD) and analyzed with one-way analysis of variance (ANOVA), followed by Dunnett’s or Tukey’s test for multiple comparison using GraphPad Prism 8.0 (Dotmatics). Differences between groups at *p* < 0.05 were considered as statistically significant.

## Supporting Information Available

Additional experimental methods; DFT, UV–vis, and antibacterial test of different metal atoms on g-C_3_N_4_; TEM of g-C_3_N_4_ bulk and nanosheets; XRD, XPS, H_2_O_2_ generation, **·**OH detection, multidrug resistance, and cell viability of Cu-*g*-C_3_N_4_; wound image and H&E/Masson tissue staining after treatment by Cu-*g*-C_3_N_4_/PCL); and Cu K-edge EXAFS fits and Gibbs free energy value (DOCX)
