# Supplementary material for: Single-Atom Cu Anchored on Carbon Nitride as a Bifunctional Glucose Oxidase and Peroxidase Nanozyme for Antibacterial Therapy
Source: ACS Nano. 2025 Mar 14;19(11):10816–28. doi: 10.1021/acsnano.4c12348 (PMC11948616; doi:10.1021/acsnano.4c12348)
Supplement: Supplementary file 1 — nn4c12348_si_001.docx [file nn4c12348_si_001.docx]

**Supporting Information**

**Single-atom Cu anchored on carbon nitride as a bifunctional glucose oxidase and peroxidase nanozyme for antibacterial therapy**

Fan Wu^1,2,3^, Yaran Wang^1,2,3^, Yuanfeng Li^1^, Linqi Shi^3,^*, Lu Yuan^2^, Yijin Ren^4^, Henny C. van der Mei^2,^*, and Yong Liu^1,3,^*

1. Translational Medicine Laboratory, the First Affiliated Hospital of Wenzhou Medical University, Wenzhou, Zhejiang 325035, China
2. University of Groningen and University Medical Center Groningen, Department of Biomaterials & Biomedical Technology, Antonius Deusinglaan 1, 9713 AV Groningen, The Netherlands
3. State Key Laboratory of Medicinal Chemical Biology, Key Laboratory of Functional Polymer Materials, Ministry of Education, Institute of Polymer Chemistry, College of Chemistry, Nankai University, Tianjin 300071, China
4. University of Groningen and University Medical Center Groningen, Department of Orthodontics, Hanzeplein 1, 9700 RB Groningen, The Netherlands

Corresponding authors: Yong Liu ([y.liu@nankai.edu.cn](mailto:y.liu@nankai.edu.cn)), Henny C. Van der Mei (h.c.van.der.mei@umcg.nl), and Linqi Shi (shilinqi@nankai.edu.cn)

**EXPERIMENTAL SECTION**

**Evaluation of ROS Generation by Detection of ·OH**. Terephthalic acid (TA) was used for measuring **·**OH, using 2-hydroxy terephthalic acid (TAOH) possessing a unique fluorescence peak at 426 nm under excitation of 312 nm.^1^ Briefly, NaOH (20 mg, 0.5 mmol) was dissolved in ultrapure water (50 mL), then TA (24 mg, 0.145 mmol) was added to the solution. The resulting mixture was stirred for 2 h till the complete dissolution of TA. Cu-g-C_3_N_4_ (0.1 g) and glucose (12 mg) were added to a 3 mL TA solution. The resultant mixture was subjected to light illumination for 30 min (*λ* ≥ 420 nm, 0.2 W cm^-2^), and the fluorescence intensity (ex/em: 312 nm/426 nm) was recorded on a LightCycler® 96 fluorescence spectrometer.

**Inhibition Zone of Cu-g-C_3_N_4_/PCL Nanofibers.** Zone of inhibition was used for evaluating the antibacterial performance of Cu-g-C_3_N_4_/PCL nanofibers. Briefly, 50 μL of *S. aureus* Xen36 suspension (10^7^ bacteria mL^-1^) containing glucose (4 mg mL^-1^) was spread on a TSB agar plate. Electrospun nanofibrous membranes containing different amounts of Cu-g-C_3_N_4_ nanosheets in PCL (0, 5, 25, and 50 µg mg^-1^) were cut into circular disk shapes (Φ_cd_ = 10 mm), and gently placed on the center of the TSB agar and illuminated for 30 min (λ ≥ 420 nm, 0.2 W cm^-2^) before incubating in humidified air at 37 ^o^C for 18 h. The antibacterial activity of the nanofibers was evaluated by measuring the diameters of its zone of inhibition.

**Tissue Cell Culture, Growth Condition and Harvesting.** L929 cells (mouse fibroblast cells) were cultured in RPMI 1640 complete medium (ThermoFisher Scientific, Inc., Carlsbad, CA) supplemented with 10% fetal bovine serum (FBS, Gibco, Shanghai, China), 100 U mL^-1^ penicillin (Genview, Beijing, China) and 100 μg mL^-1^ streptomycin (Solarbio, Beijing, China). Fibroblasts cells were incubated in a humidified atmosphere of 5% CO_2_ at 37 °C, when the cells reached 70-90% confluency, cells were detached from the cell-culture flask by EDTA-trypsin for 2 min, collected by centrifugation at 1000 rpm for 5 min, and re-suspended in fresh cell culture medium for the determination of the cytotoxicity.

**Cytotoxicity of Cu-g-C_3_N_4_ Nanosheets.** Fibroblasts (1 × 10^5^ mL^-1^) cells RPMI complete medium were seeded in 96-well plates and cultured in a humidified 5% CO_2_ incubator at 37 °C for 24 h. After 24 h, the culture medium was refreshed with a medium containing Cu-g-C_3_N_4_ nanosheets at different concentrations (0, 6.3, 12.5, 25, 50, and 100 µg mL^-1^), glucose (4 mg mL^-1^) and illuminated with light for 30 min (*λ* ≥ 420 nm, 0.2 W cm^-2^). Then the cells were incubated with CCK-8 in order to determine the metabolic activity in a humidified 5% CO_2_ incubator at 37 °C for 1 h. The metabolic activity of the cells was determined by measuring the optical density (OD) at 450 nm on an Epoch2 microplate reader. The metabolic activity was used as a measure for viability and calculated by Eq. S1. The cells exposed to PBS were used as control.

Cell viability (%) = (OD value of experimental group)/(OD value of control group)×100% (S1)

**Supplementary Figures**


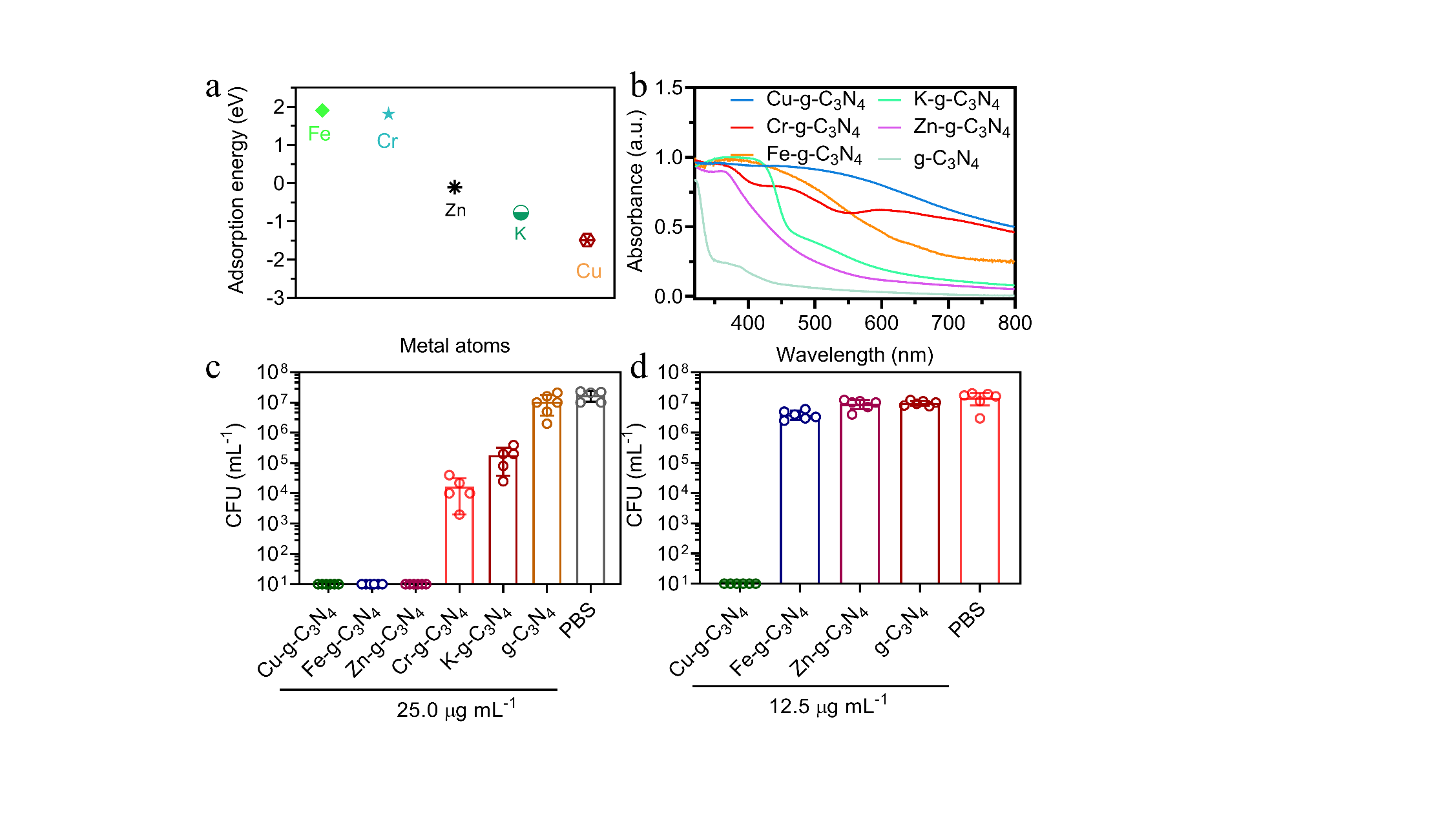


**Figure S1. Adsorption energy, UV-vis spectra, and antibacterial activity of different metal atoms (Fe, Cr, Zn, K, and Cu) on the g-C_3_N_4_.** (a) Adsorption energy of different metal atoms (Fe, Cr, Zn, K, and Cu) on the g-C_3_N_4_ structure. Theoretical calculations of adsorption energy were carried out with computational quantum mechanical modelling. The adsorption energy was calculated as the difference between the total energy of g-C_3_N_4_ nanosheets with adsorbed metal and the energy of metal and the energy of g-C_3_N_4_. (b) UV-vis spectra of pure g-C_3_N_4_ nanosheet powder and g-C_3_N_4_ nanosheet powder anchored with Cu, Cr, K, Fe, and Zn. Cu-g-C_3_N_4_ nanosheets exhibit a higher light absorption property than g-C_3_N_4_ nanosheets. Antibacterial activity of g-C_3_N_4_ nanosheets anchored with different metals against *Staphylococcus aureus* Xen36. Killing efficacy on planktonic bacteria (10^7^ bacteria mL^-1^) of different concentrations (25.0 µg mL^-1^ (c) and 12.5 µg mL^-1^ (d)) of g-C_3_N_4_ nanosheets anchored with different metals (Cu, Cr, K, Fe, and Zn) (glucose concentration: 4 mg mL^-1^, light: λ ≥ 420 nm, 0.2 W cm^-1^, 30 min). Cu-g-C_3_N_4_ (12.5 µg mL^-1^) nanosheets could eradicate bacteria and kill the bacteria than other metal-g-C_3_N_4_.


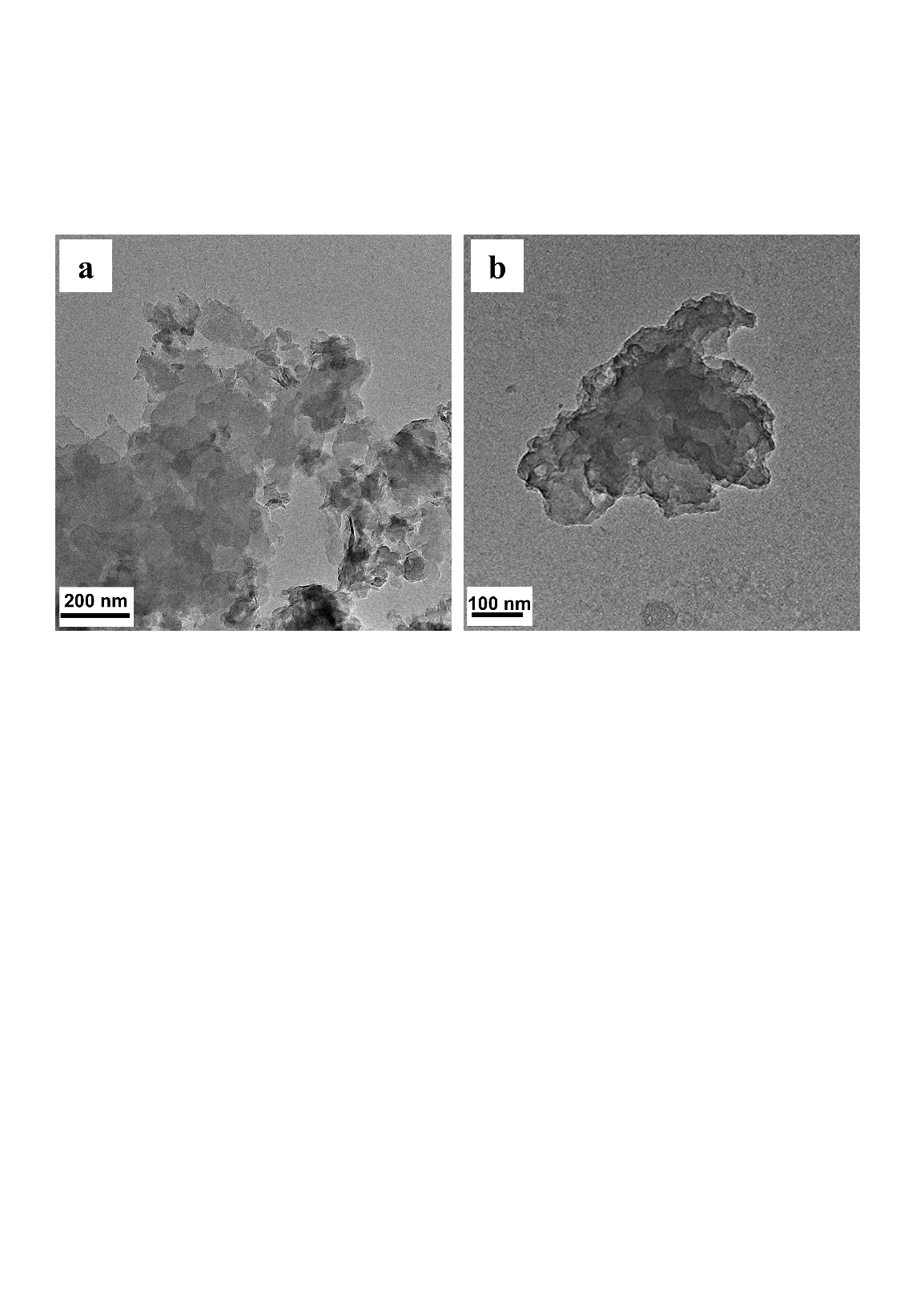


**Figure S2. The morphological characterization of g-C_3_N_4_.** (a) TEM of bulk g-C_3_N_4_ and (b) g-C_3_N_4_ nanosheets after treatment by sulfuric acid to confirm the successful synthesis of g-C_3_N_4_ nanosheets.


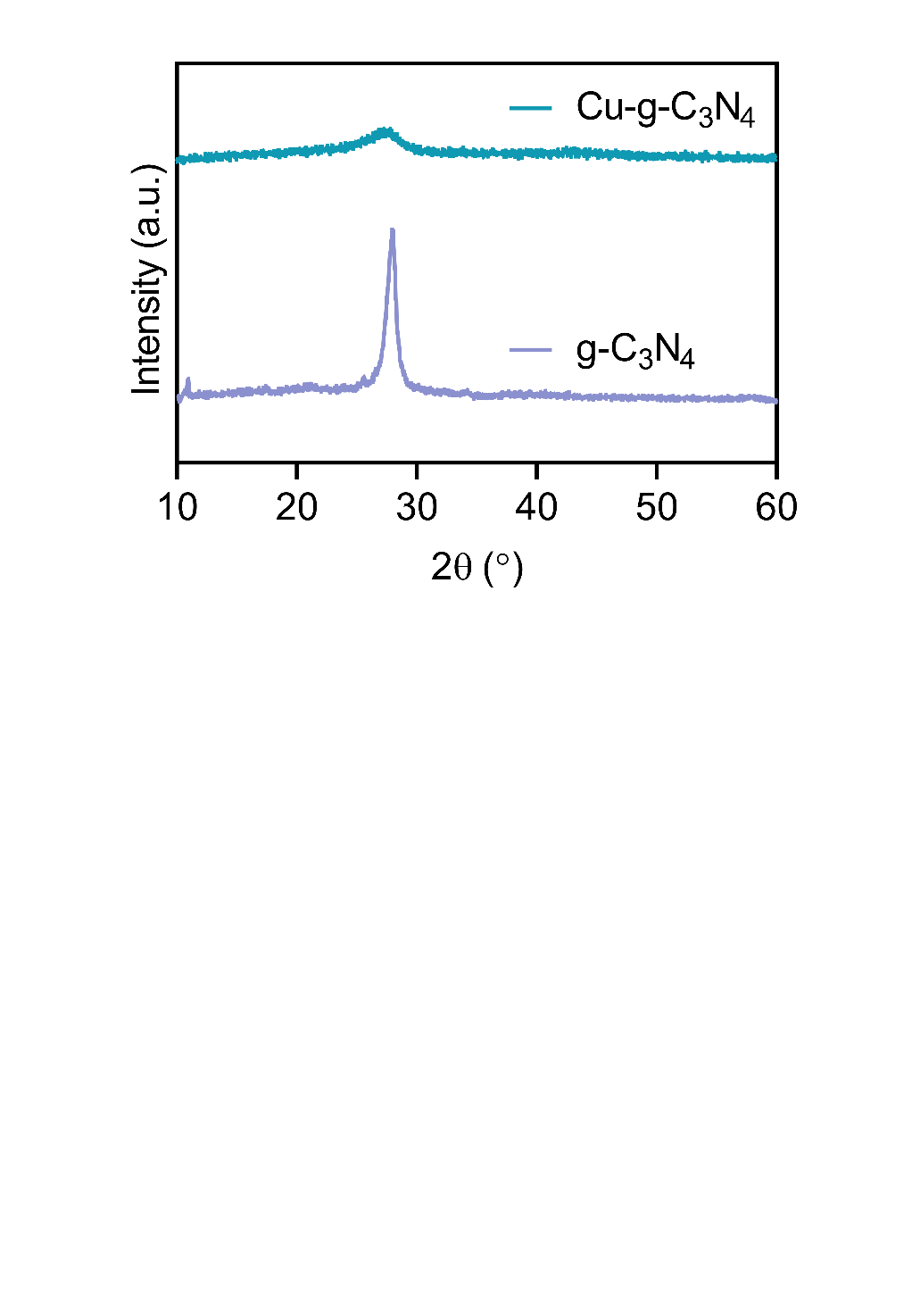


**Figure S3. X-ray powder diffraction spectra of g-C_3_N_4_ and Cu-g-C_3_N_4_ nanosheets**


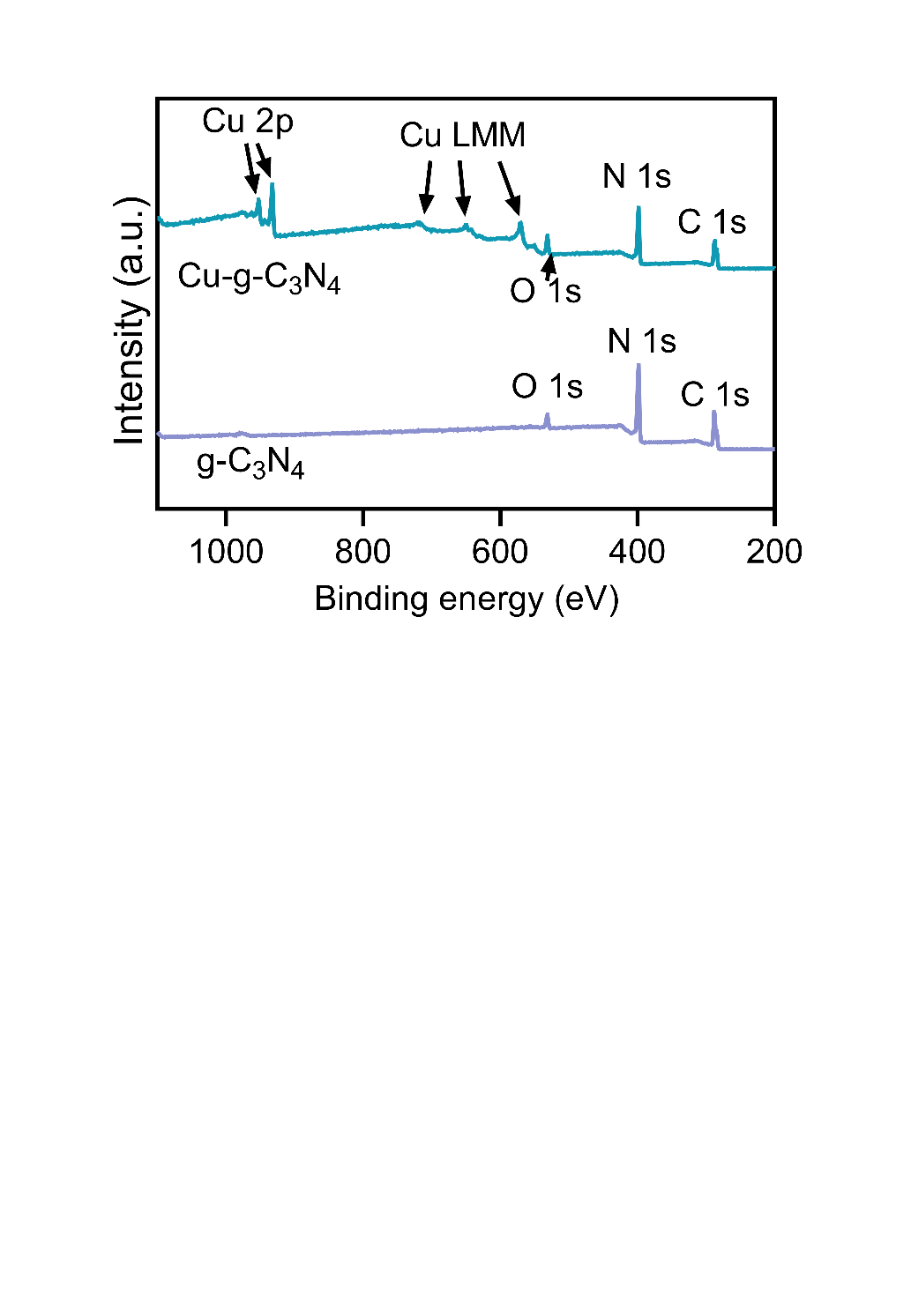


**Figure S4. XPS** **wide scan spectra of g-C_3_N_4_ and Cu-g-C_3_N_4_ nanosheets indicating that Cu was introduced into g-C_3_N_4_ nanosheets.**


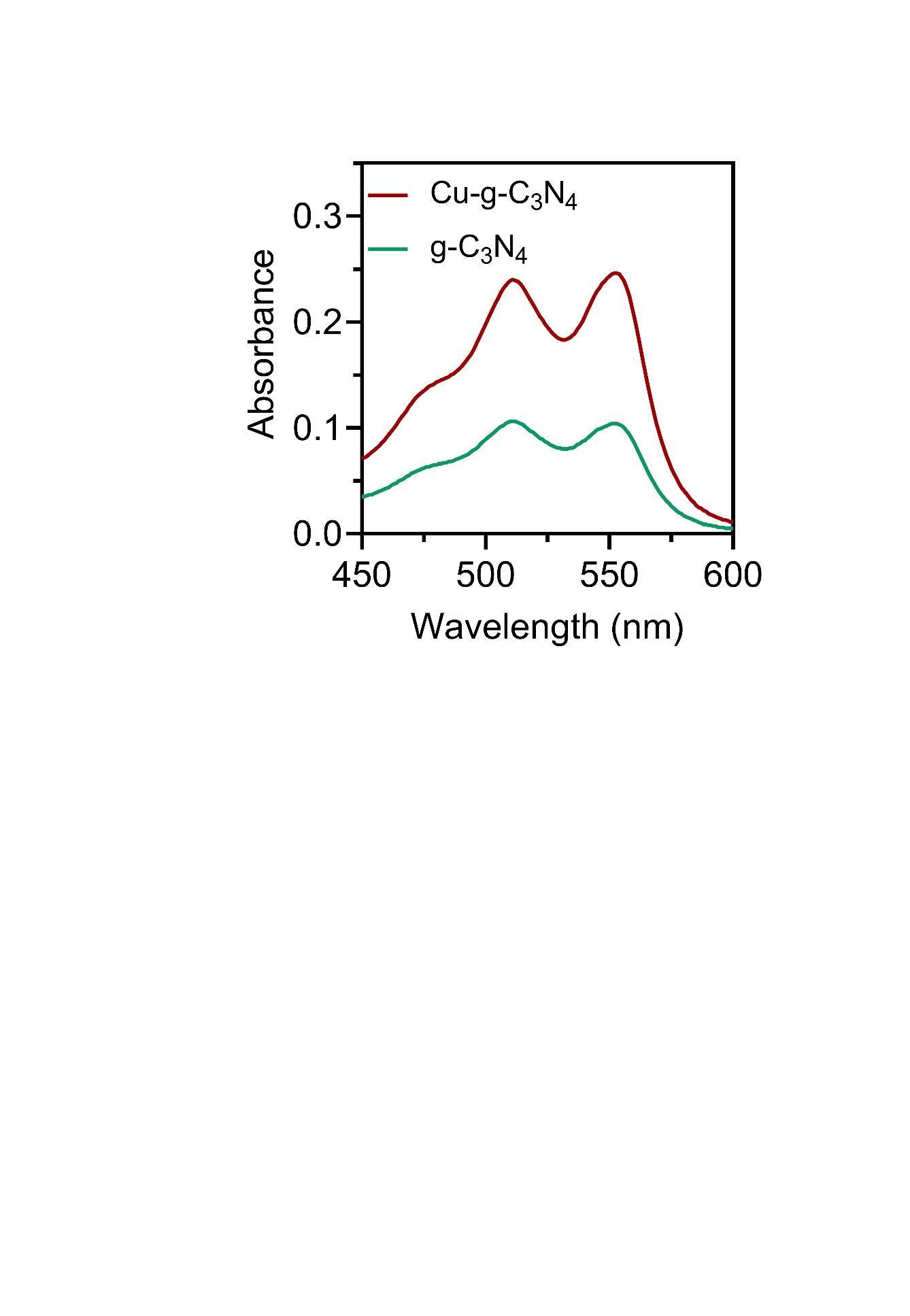


**Figure S5.** UV-vis spectra of H_2_O_2_ generation from glucose oxidation (glucose concentration: 4 mg mL^-1^) using g-C_3_N_4_ nanosheets and Cu-g-C_3_N_4_ nanosheets (50 µg mL^-1^) in phosphate buffer (pH 7) with visible light irradiation for 30 min (λ ≥ 420 nm, 0.2 W cm^-1^) using N,N-diethyl-l,4-phenylenediammonium sulfate (DPD) and peroxidase (POD).The baseline has been corrected.


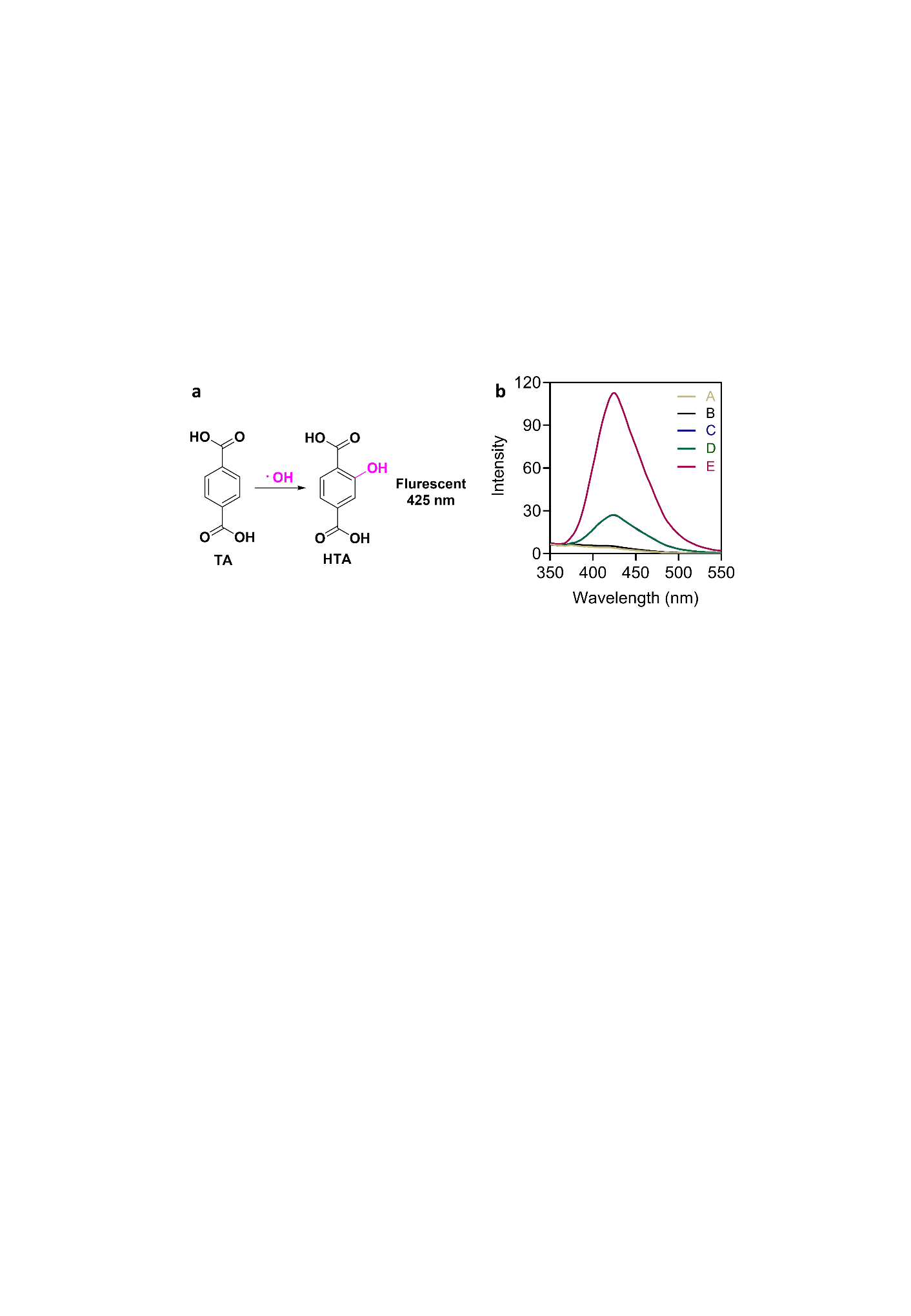


**Figure S6. Detection of** •**OH.** (a) Scheme of •OH detection using terephthalic acid (TA) as a probing molecule. (b) Fluorescence intensity under excitation of 312 nm. The fluorescence peak of HTA is around 426 nm. Treatment of Cu-g-C_3_N_4_ nanosheets with glucose and light ((λ ≥ 420 nm, 0.2 W cm^-2^)) showed the highest peak corresponding with the amount of •OH generated. A: Control; B: Cu-g-C_3_N_4_/light; C: Cu-g-C_3_N_4_/glucose; D: Light/glucose; E: Cu-g-C_3_N_4_/glucose/light.


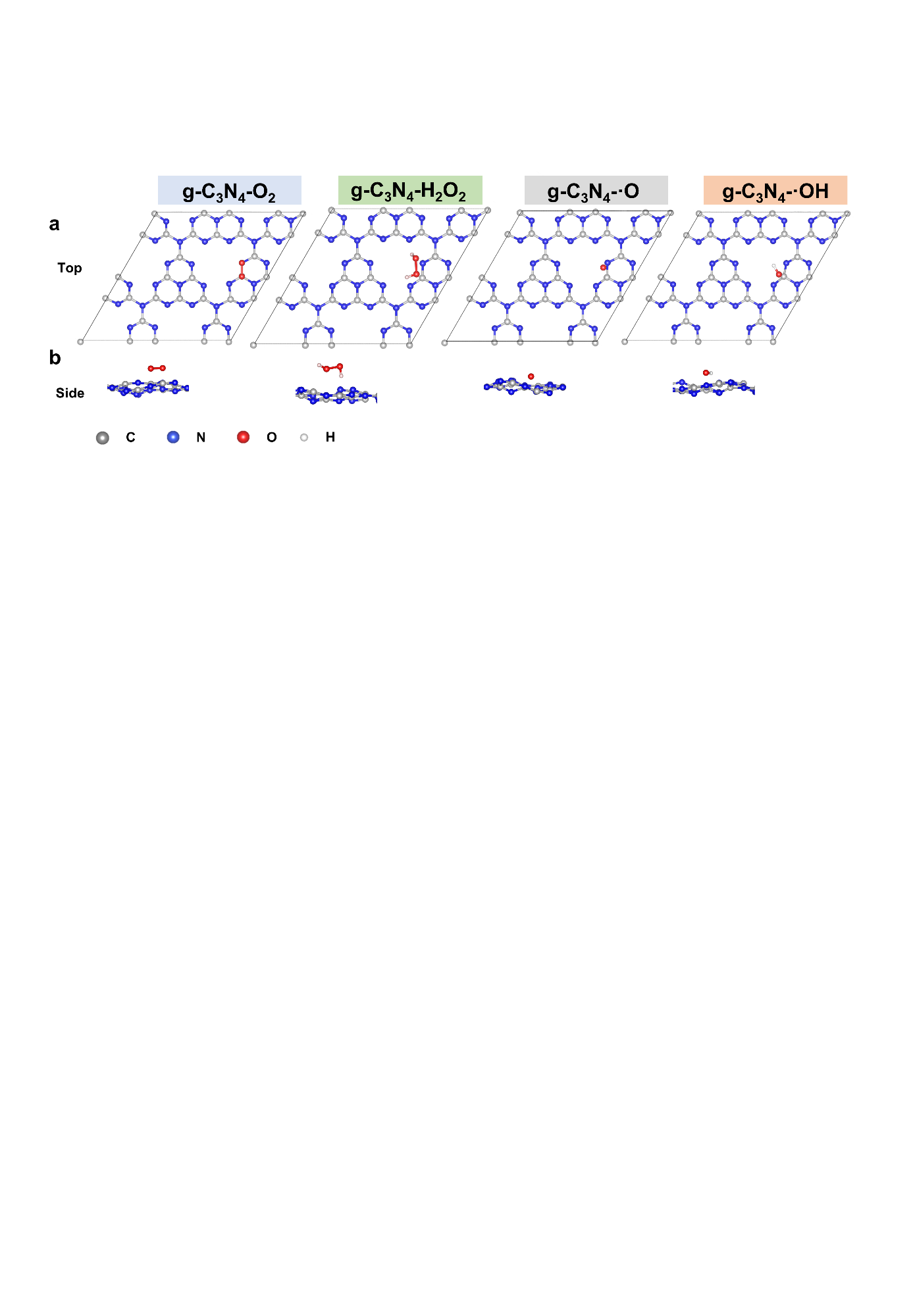


**Figure S7. Density functional theory (DFT) of oxygen species adsorbed on g-C_3_N_4_ nanosheets.** Top (a) and side (b) views of the structure of O_2_, H_2_O_2_, •O, and •OH adsorbed on g-C_3_N_4_ nanosheets


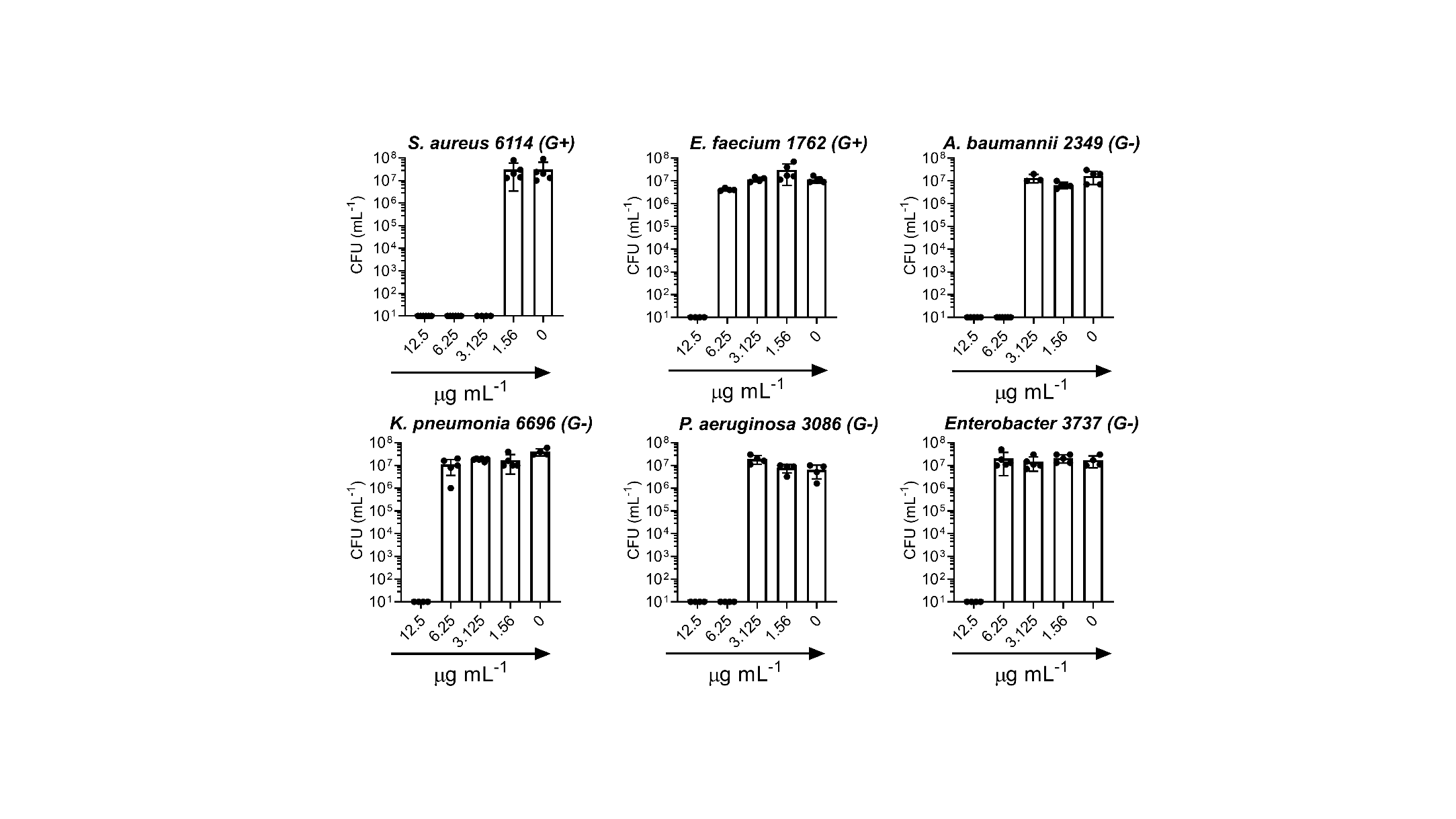


**Figure S8.** **Antibacterial activity of Cu-g-C_3_N_4_ nanosheets against** **multi-drug resistant Gram-positive (G^+^) and Gram-negative (G^-^) bacteria**. Killing efficacy on planktonic bacteria (10^7^ bacteria mL^-1^) of different concentrations of Cu-g-C_3_N_4_ nanosheets (12.5, 6.25, 3.125, 1.56, and 0 µg mL^-1^), glucose concentration: 4 mg mL^-1^, light: λ ≥ 420 nm, 0.2 W cm^-2^, 30 min.


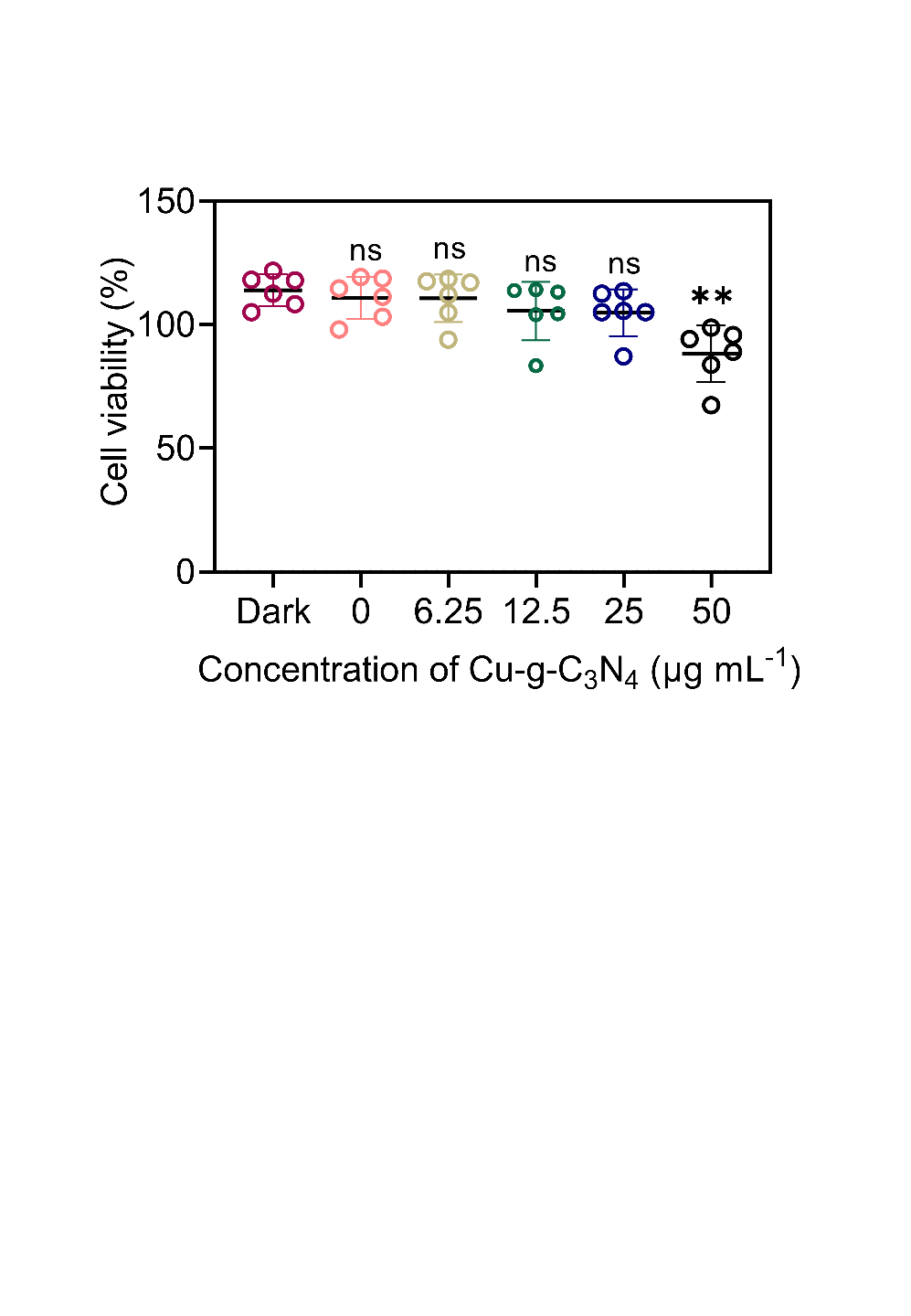


**Figure S9. Relative cell viability of L929 cells incubated with different concentrations of Cu-g-C_3_N_4_ nanosheets.** Statistical significance of differences with respect to PBS treatment (n=6) indicates as **p < 0.01 and *ns* stands for no significant difference. Cell viability is expressed relative to cells not exposed to the Cu-g-C_3_N_4_ nanosheets, light, or glucose in the RPMI medium, which are put on 100%.


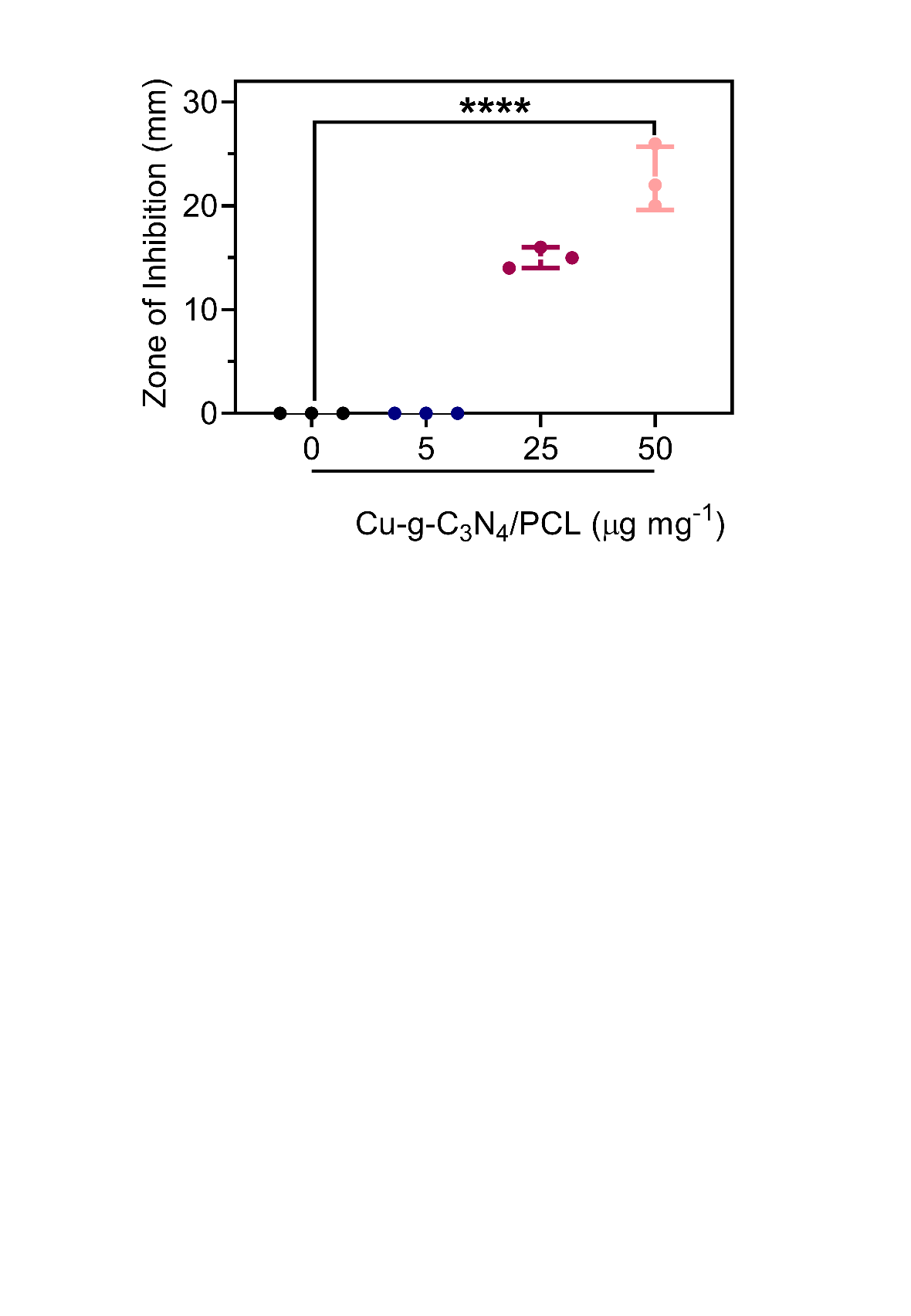


**Figure S10. Zone of inhibition of Cu-g-C_3_N_4_/PCL nanofibers on an agar plate inoculated with *S. aureus* Xen36.** The zone of inhibition was measured for different concentrations of the Cu-g-C_3_N_4_ on the nanofibers (0, 5, 25, 50 µg mg^-1^). Statistical significance differences with respect to PBS (n=6) **** indicates p < 0.0001.


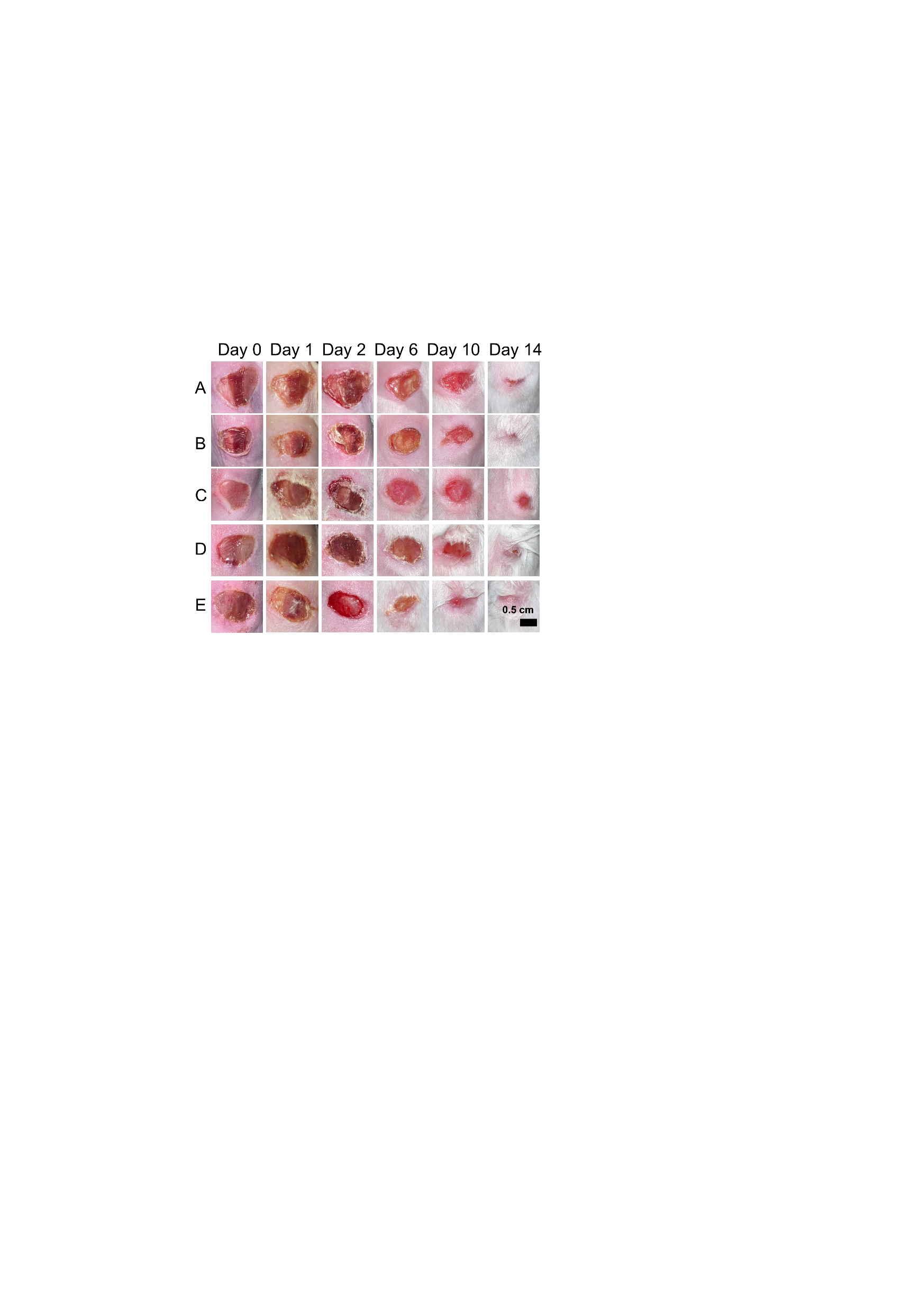


**Figure S11. Images of the** **wound area with different treatments over a 14-day observation period.** The images obtained on days 0, 1, 2, 6, 10, and 14 after different treatments. The A, B, C, D, and E represent mice treated with PBS, ciprofloxacin, Cu-g-C_3_N_4_/PCL/light, Cu-g-C_3_N_4_/PCL/glucose, and Cu-g-C_3_N_4_/PCL/glucose/light, respectively.


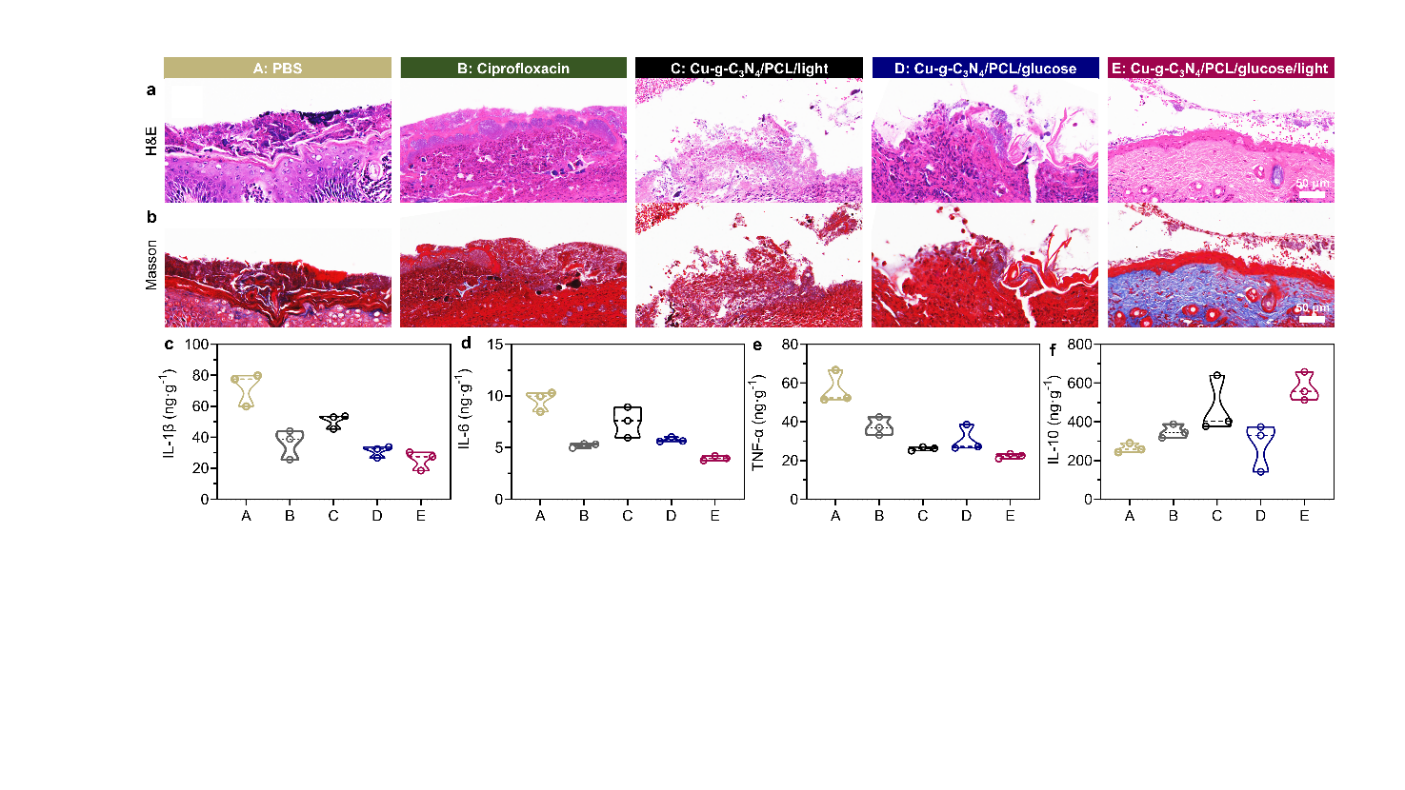


**Figure S12. Qualitative and quantitative analysis of inflammatory activities in tissue from infected wounds after treatment with Cu-g-C_3_N_4_ with and without glucose and light.**

(a) Hematoxylin and eosin (H&E) and (b) Masson tissue staining on day 14 after different treatments. The H&E stains the nucleus (blue) and cytoplasm (red) to observe inflammatory cells. For Masson staining, collagen fibers can be stained to blue, and muscle fibers are stained to red. (c-f) Expression of inflammatory cytokines (IL-1β, IL-6, TNF-α, and IL-10) in wound tissues on day 2. Data are presented as means ± standard deviations over three mice in each group.


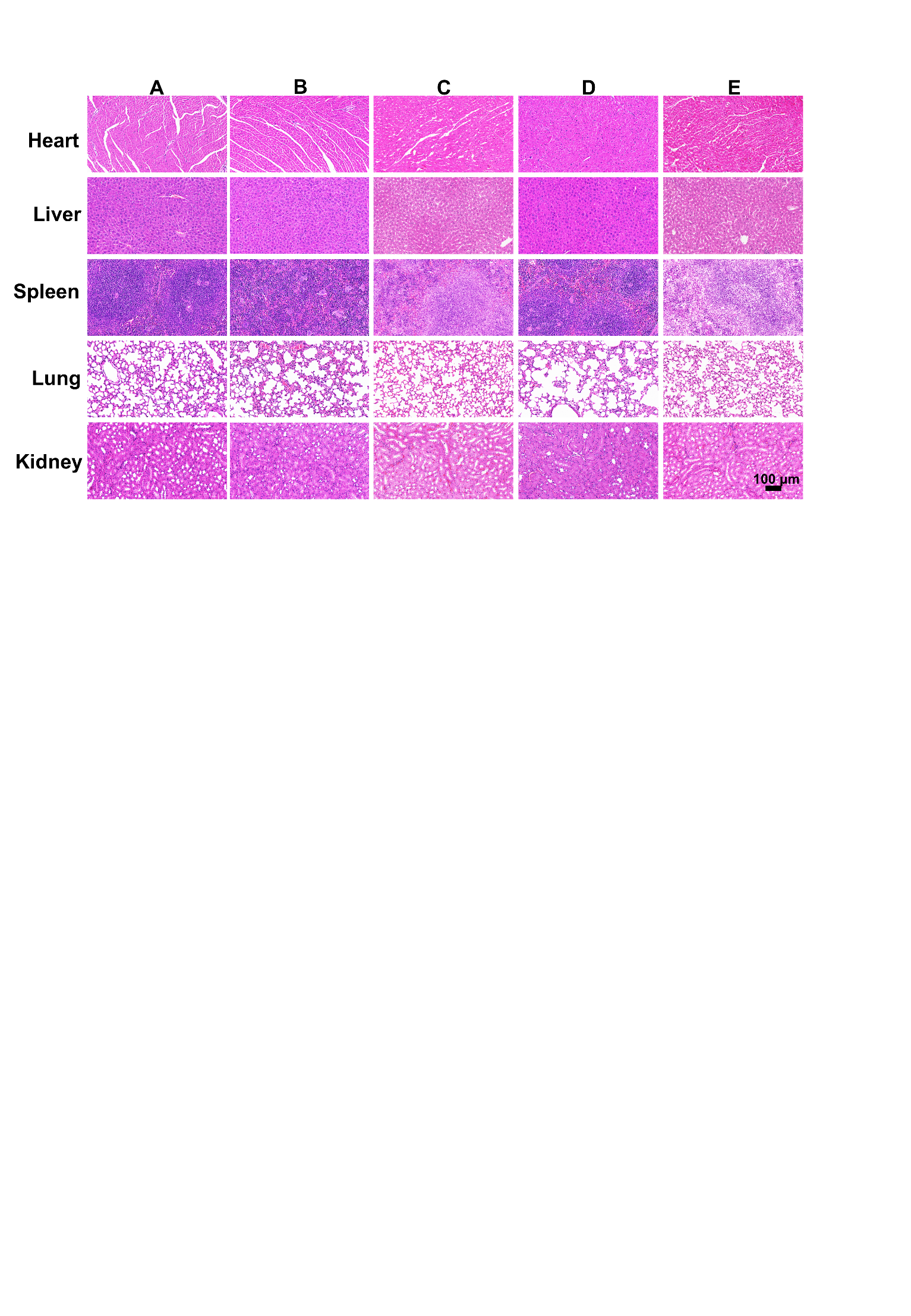


**Figure S13. H&E staining of sections of the major organ in mice.** H&E staining of heart, liver, spleen, lung, and kidney sections harvested from mice after various treatments on day 14. Bar represents 100 μm. The A, B, C, D, and E represent mice treated with PBS, ciprofloxacin, Cu-g-C_3_N_4_/PCL/light, Cu-g-C_3_N_4_/PCL/glucose, and Cu-g-C_3_N_4_/PCL/glucose/light, respectively.**Supplementary Table**

**Table S1. Structural parameters obtained from the** **Cu K-edge EXAFS fits.**

| sample | bond | CN | R (Å) | σ^2^ (Å^2^) | △E (eV) | χ_ν_^2^ / R factor |
| --- | --- | --- | --- | --- | --- | --- |
| Cu-g-C_3_N_4_ | Cu-N | 4.2 | 1.92 (±0.013) | 0.008 (±0.0019) | -4.6（±2.28） | 41.0/0.0059 |

**Table S2.** **Gibbs free energy of •H_2_O_2_, •O or •OH on g-C_3_N_4_ and Cu-g-C_3_N_4_ nanosheets.**

|  | •H_2_O_2_ (eV) | •O (eV) | •OH (eV) |
| --- | --- | --- | --- |
| g-C_3_N_4_ | -1.77 | 6.12 | 1.13 |
| Cu-g-C_3_N_4_ | -2.05 | 4.18 | -0.94 |

**REFERENCE**

(1) Wang, Z.; Zhang, Y.; Ju, E.; Liu, Z.; Cao, F.; Chen, Z.; Ren, J.; Qu, X. Biomimetic Nanoflowers by Self-Assembly of Nanozymes to Induce Intracellular Oxidative Damage against Hypoxic Tumors. *Nat. Commun.* **2018**, *9*, 3334.
